# Lack of GPNMB Is Associated With Altered Lipid and Glucose Metabolism and Disrupted Diurnal Hepatic Glycogen Regulation

**DOI:** 10.1096/fj.202504363R

**Published:** 2026-03-09

**Authors:** Eliz Maria de Oliveira Furtado, Lina Jegodzinski, Juliano Jefferson da Silva, Ana Flávia Tostes, Lucas dos Santos, Pietra Souza Barsanele, Beatriz Santana‐Lima, Luciana Chagas Caperuto, Mery Natali Silva Abreu, Darko Castven, Andrea Schenk, Danusa Soares Dias, Jens U. Marquardt, Maristela Oliveira Poletini, Leonardo Vinícius Monteiro de Assis, Maria Nathália Moraes

**Affiliations:** ^1^ Laboratório de Cronobiologia Molecular, Departamento de Ciências Biológicas, Instituto de Ciências Ambientais Químicas e Farmacêuticas Universidade Federal de São Paulo São Paulo Brazil; ^2^ Programa de Pós‐graduação Em Fisiologia, Instituto de Biociências Universidade de São Paulo São Paulo Brazil; ^3^ Department of Medicine 1 University Medical Center Schleswig‐Holstein, Campus Lübeck Lübeck Germany; ^4^ Laboratório de Neurobiologia, Departamento de Fisiologia e Biofísica, Instituto de Ciências Biomédicas Universidade de São Paulo São Paulo Brazil; ^5^ Laboratório de Fisiologia Metabólica, Departamento de Ciências Biológicas, Instituto de Ciências Ambientais Químicas e Farmacêuticas Universidade Federal de São Paulo São Paulo Brazil; ^6^ Departamento de Gestão Em Saúde, Escola de Enfermagem Universidade Federal de Minas Gerais Belo Horizonte Brazil; ^7^ Department of Surgery University Medical Center Schleswig‐Holstein, Campus Lübeck Lübeck Germany; ^8^ Laboratório de Fisiologia Do Exercício, Escola de Educação Física, Fisioterapia e Terapia Ocupacional Universidade Federal de Minas Gerais Belo Horizonte Brazil; ^9^ Laboratório de Endocrinologia e Metabolismo, Instituto de Ciências Biológicas Universidade Federal de Minas Gerais Belo Horizonte Brazil; ^10^ Institute of Neurobiology, Center of Brain Behavior & Metabolism University of Lübeck Lübeck Germany; ^11^ University Hospital Schleswig‐Holstein, Campus Lübeck Lübeck Germany; ^12^ Department of Chemistry and Molecular Biology University of Gothenburg Gothenburg Sweden; ^13^ Wallenberg Centre for Molecular and Translational Medicine University of Gothenburg Gothenburg Sweden

**Keywords:** circadian disruption, circadian rhythm, hepatic metabolism, high‐fat diet

## Abstract

Higher serum levels of GPNMB are linked to type 2 diabetes mellitus (T2DM) and metabolic dysfunction‐associated steatotic liver disease (MASLD). Disruption of circadian rhythms also influences the development and progression of MASLD. In this study, we investigated how GPNMB modulates hepatic glycogen metabolism and its potential interaction with the hepatic circadian clock. Male DBA/2 J mice, either wild‐type (GP^+^) or carrying an inactivating *Gpnmb* mutation (GP^−^), were fed a high‐fat diet (48.4% fat) supplemented with 30% fructose in drinking water for 12 weeks. Despite similar weight gain, GP^−^ mice displayed greater global fat mass accumulation and elevated serum triglyceride and cholesterol levels. Surprisingly, GP^−^ mice showed improved glucose tolerance, whereas GP^+^ mice developed impaired glycemic control. Indirect calorimetry under thermoneutral conditions (30°C) revealed loss of diurnal rhythmicity in energy expenditure (EE) in GP^−^ mice, which was independent of food intake. Despite its preserved rhythms, hepatic clock gene expression in GP^−^ mice showed increased MESOR (e.g., *Per1*, *Per2*, and *Nr1d1*) and increased amplitude (e.g., *Nr1d1*), indicating higher expression levels throughout the day. GPNMB deficiency further impaired hepatic glycogen storage dynamics, which was attributed to reduced AKT phosphorylation (indicative of defective insulin signaling), reduced FOXO1 phosphorylation, and increased PEPCK‐M. Translating our findings to human MASLD patients, *GPNMB* expression obtained from liver biopsies showed a clear increase across MASLD progression. Importantly, patients with metabolic dysfunction‐associated steatohepatitis (MASH) and diabetes who received anti‐diabetic treatment showed a reduction in hepatic *GPNMB* expression. Collectively, our findings suggest that GPNMB plays a role in metabolic adaptation to obesogenic diets, as a *Gpnmb* loss‐of‐function model reveals an association with impaired hepatic insulin signaling and glycogen metabolism despite improved systemic glucose tolerance in mice, whereas hepatic GPNMB upregulation correlates with MASLD progression in humans.

## Graphical abstract

Loss of GPNMB function in mice fed a high‐fat/high‐fructose diet aggravated adiposity and dyslipidemia while preserving systemic glucose tolerance. This phenotype was associated with impaired hepatic AKT–FOXO1 signaling, increased NF‐κB activation, altered glycogen metabolism, and rhythmic disturbances in energy expenditure and clock gene expression. In humans, hepatic GPNMB expression increased with MASLD progression and decreased following antidiabetic treatment. Together, these results suggest a role for GPNMB in metabolic adaptation and disease progression. Graphical Abstract created with BioRender. De assis, L. (https://BioRender.com/11gyv3v) is licensed under CC BY 4.0.

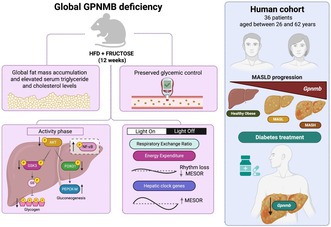

## 1 Introduction

Organisms are strongly influenced by predictable environmental variations that occur throughout the day, especially the light/dark and temperature cycles [1, 2]. To predict and adjust physiological processes to environmental changes, mammals have evolved internal biological clocks [3]. Biochemical, physiological, and behavioral processes—such as enzyme activity, energy metabolism, immune function, and sleep–wake cycles—follow daily rhythmic patterns that recur roughly every 24 h, called circadian rhythms [4, 5]. These rhythms are driven by an endogenous molecular mechanism that aligns the body's internal biological clock with external environmental signals, with light–dark cycles being the strongest synchronizer of the central circadian clock [5, 6]. At the cellular level, this temporal regulation is controlled by a series of feedback loops involving clock genes and proteins, which coordinate the expression of target genes and organize physiological rhythms [7]. These loops are the basis of circadian molecular clocks found in all nucleated cells. The liver exhibits a series of circadian biological processes that are vital for regulating systemic metabolism by controlling glucose, lipid, protein, and cholesterol metabolism [8, 9, 10]. Transcriptomic analyses show that circadian regulation drives rhythmic expression of approximately 10%–15% of liver genes [11, 12, 13].

Metabolic disorders such as obesity, insulin resistance, dyslipidemia, and hepatic steatosis have become widespread globally, primarily caused by factors like excessive consumption of high‐fat and high‐carbohydrate diets. These dietary patterns disrupt energy homeostasis, elevate systemic inflammation, and impair peripheral circadian regulation [13, 14, 15, 16, 17, 18, 19]. Metabolic dysfunction‐associated steatotic liver disease (MASLD) has emerged as the most prevalent chronic liver disease over the last few decades [20, 21], affecting approximately 38% of the global adult population [22, 23] and accompanied by complications such as type 2 diabetes mellitus (T2DM), cardiovascular and renal diseases [24, 25, 26]. The highest prevalence of MASLD is observed in people with obesity (75%) or T2DM (69%) [22, 27]. The coexistence of obesity and T2DM can markedly accelerate MASLD progression [22, 26, 27].

The protein GPNMB (glycoprotein non‐metastatic melanoma protein B), a transmembrane glycoprotein, has been associated with several diseases, including obesity, neuroinflammation, MASLD, T2DM, and cancer progression. GPNMB is expressed in immune cells such as macrophages, monocytes, and microglia [28, 29, 30, 31, 32]. In patients with obesity, T2DM, and MASH, there is an increase in plasma GPNMB expression, directly correlating with body mass index (BMI) [29]. Hepatocyte‐specific overexpression of GPNMB induces lipogenesis in white adipose tissue, exacerbating insulin resistance and obesity [29]. Diets rich in fat, fructose, or cholesterol, or deficient in choline, increase hepatic *Gpnmb* expression [8, 33, 34, 35]. Therefore, GPNMB emerges as a key element in the pathophysiology of metabolic disorders [29, 30].

Despite the known involvement of GPNMB in metabolic diseases, its effects on the liver and circadian rhythms are still poorly explored. Our study aimed to investigate the role of GPNMB in regulating liver metabolism and its potential influence on circadian rhythms. Our findings reveal that GPNMB plays an essential role in glucose metabolism, directly interfering with the insulin pathway and hepatic glycogen storage in mice. Despite the preservation of the core molecular machinery of the hepatic clock in the absence of GPNMB, the livers of these mice display a disruption of critical rhythmic parameters, leading to increased glycogen accumulation in the resting phase. These alterations were associated with a time‐dependent reduction in AKT phosphorylation and a reduction in FOXO1 phosphorylation with a consequent increase in PEPCK‐M expression. Furthermore, analysis of human liver samples with MASLD revealed that *GPNMB* expression increases during MASLD progression, that is, from a simple steatosis to Metabolic dysfunction‐associated steatohepatitis (MASH), being positively associated with glycated hemoglobin. Notably, patients with MASLD undergoing anti‐diabetic treatment exhibited a decrease in *GPNMB* levels, indicating that hepatic *GPNMB* responds to diabetic therapy and may serve as a potential marker of MASLD progression and development.

## 2 Materials and Methods

### 2.1 Mouse Strain and Housing Condition

We used the DBA/2 J‐GPNMB strain as a model to investigate the effects of the functional absence of global GPNMB on liver metabolism. DBA/2 J mice carry a spontaneous mutation in the *Gpnmb* gene that results in a non‐functional GPNMB protein (GP^−^) (Jackson Laboratory JAX:000671) [36]. As control, we used DBA/2 J‐GPNMB+ (GP^+^) mice, which are coisogenic to the GP^−^ line and differing only at the *Gpnmb* locus, while sharing the same DBA/2 J genetic background (Jackson Laboratory JAX:007048). The experimental procedures were conducted in accordance with national and international ethical guidelines and regulations and were approved by the Ethics Committee for the Use of Animals (ICB/USP N°. 7 699 101 023). Male mice between 2 and 3 mo76nths old were randomly housed in groups of two to three per cage at 25°C ± 1°C under a 12:12 h light/dark cycle (12:12 LD). Lighting was provided by LED lamps (400 lx, spectral range of 420–750 nm), with light on at 7 am (zeitgeber time—ZT0) and off at 7 pm (ZT12). Mice were fed either a normolipidic diet (ND; Nuvilab, Brazil—composed of 8.53% fat, 52.99% carbohydrates, and 38.47% protein) or a high‐fat diet (HFD), both provided *ad libitum*, with free access to water. The HFD consisted of 48.4% kilocalories from fat, 30.1% from carbohydrates, and 21.5% from protein (PragSoluções, Brazil), and it was combined with the oral administration of a 30% fructose solution (Synth, Brazil) provided *ad libitum* instead of tap water. Animals in the HFD group were maintained on this dietary regimen for up to 12 weeks. Body weight, food, and water intake were tracked over the 12 weeks. Mice were weighed twice weekly in the morning using a digital scale, and food and water consumption were measured per cage and reported as weekly averages per animal.

### 2.2 Body Composition by Magnetic Resonance

At the end of the 8th and 12th weeks of the diet protocol, body composition was assessed by nuclear magnetic resonance (Minispec LF50, Bruker, Germany) to measure fat mass, lean mass, and body fluids. The procedure was conducted on conscious animals, gently restrained in an acrylic holder, without needing anesthesia, and took about 30 s, following previously reported methods [37].

### 2.3 Glucose Tolerance Tests

The intraperitoneal glucose tolerance test (IPGTT) was performed at Weeks 8 and 12 of the diet protocol after an 8 h fast (ZT2‐10). Blood glucose was measured from tail vein sampling at baseline (0 min) and at 5, 15, 30, 60, 90, and 120 min after glucose injection (2 g/kg, i.p.). The test was carried out at the end of the resting phase (ZT10‐ZT12), using an Optium Xceed glucometer and Optium reagent strips (Medisense, UK).

### 2.4 Measurement of the Metabolic Rate

Indirect calorimetry was performed using the FoxBox Respirometry System (Sable Systems, USA) to measure oxygen consumption (VO_2_) and carbon dioxide production (VCO_2_), which reflect in vivo metabolic rate and substrate utilization. Measurements were taken after 8 and 12 weeks of HFD + FRUT exposure. After a 4–6 h acclimation period in the respirometric chamber, mice were monitored for 36 h at 30°C (within the thermoneutral zone) under a 12:12 h light–dark cycle. Oxygen consumption (VO_2_) and carbon dioxide production (VCO_2_) were recorded every 30 min and normalized to body weight. The respiratory quotient (RQ = VCO_2_/VO_2_) and energy expenditure (EE) were calculated from VO_2_ and RQ values and expressed in kJ/h, according to the equations described by [38] and [39].

### 2.5 Plasma Biochemical Analysis

At the end of the 12 week diet protocol, mice were euthanized by decapitation, and blood samples were collected. Samples were transferred to EDTA‐K3‐treated microtubes (FirstLab, Brazil), centrifuged at 1200 ***g*** for 15 min at 4°C to obtain plasma, and stored at −80°C until analysis. Plasma levels of total cholesterol, triglycerides, and alanine aminotransferase (ALT) were determined by spectrophotometry on a Labmax 240 analyzer, using commercial kits (Cholesterol Liquiform—Ref. 76; ALT/GPT Liquiform—Ref. 108; Triglycerides—Ref. 87, Labtest, Brazil). A total of 200 μL of plasma was used to perform all assays. Analyses were performed at the Department of Food and Experimental Nutrition, FCFRP‐USP.

### 2.6 Quantitative Real‐Time PCR

Mice were euthanized by decapitation every 4 h, starting at ZT0 (light turned on), to analyze hepatic gene expression. Total RNA from liver fragments was extracted using TRIzol reagent, according to the manufacturer's instructions (Thermo Fisher Scientific, Waltham, MA, USA). The cDNA was synthesized from 1 μg of total RNA using Superscript III reverse transcriptase with 100 ng random hexamer primers and RNaseOUT, according to the manufacturer's instructions (Thermo Fisher Scientific, Waltham, MA, USA). The genes of interest were quantified by real‐time PCR using 5 ng of cDNA on the QuantStudio 5 Flex system (Applied Biosystems, RRID:SCR_020240), using the SYBR Green system. The primers, designed to span exon–exon junctions, were synthesized by IDT (Coralville, IA, USA) or Thermo Fisher Scientific (Waltham, MA, USA), as listed in Table 1. Gene expression was normalized by the constitutive gene *Rpl37a*, with a final concentration of 300 nM per primer. Gene expression was quantified using the 2^–ΔΔCT^ method, as described by [40]. The lowest mean ΔCT value of the control group was used as a calibrator and subtracted from the other values (control and experimental) to obtain ΔΔCT. The relative expression of the genes was then calculated as 2^–ΔΔCT^.

**TABLE 1:** Gene access numbers, species, and sequences of primers.

Gene (acess number) and specie	Primer forward (5′–3′)	Primer backward (5′–3′)
*hGAPDH (NM_001289746.2)*	5′‐CAACGACCACTTTGTCAAGC‐3′	5′‐TCTTCCTCTTGTGCTCTTGC‐3′
*hGPNMB (NM_001005340.2)*	5′‐TGTTCCTGACAGAGACCCAGC‐3′	5′‐TTGTACACCAAGAGGGAGATCACA‐3′
*hFOXO1 (NM_002015.4)*	5′‐GAGGGTTAGTGAGCAGGTTACA‐3′	5′‐TGCTGCCAAGTCTGACGAAA‐3′
*hFBP1 (NM_000507.4)*	5′‐TGCACAGCAGTCAAAGCCAT‐3′	5′‐ACCAGCAATGCCATAGAGGTG‐3′
*hSREBPF1* (NM_001005291.3)	5′‐GACCGACATCGAAGGTGAAGT‐3′	CCAGCATAGGGTGGGTCAAA‐3′
*mBmal1* (NM_001243048.2)	5′‐AAGCTTCTGCACAATCCACAGCAC‐3′	5′‐TGTCTGGCTCATTGTCTTCGTCCA‐3′
*mGys2* (NM_145572.2)	5′‐CACATCACCACCAACGACGGA‐3′	5′‐GGCTGAGAGGGATCGGCTAAA‐3′
*mGpnmb* (NM_053110.4)	5′‐ATGGAAAGTCTCTGCGGGGT‐3′	5′‐GCACATCACGAAATCGCTTG‐3′
*mHsl* (NM_001039507.2)	5′‐GGGAGGGCCTCAGCGTTCTCACA‐3′	5′‐ATAGCACGGAGCTGGGTGAGG‐3′
*mIl6r‐α* (NM_010559.3)	5′‐GGGTGTACTCTGGCTCACAAA‐3′	5′‐CCCGTTGGTGGTGTTGATTT‐3′
*mNr1d1* (NM_145434.4)	5′‐AAGACATGACGACCCTGGAC‐3′	5′‐CCATGCCATTCAGCTTGGTAAT‐3′
*mPer1* (NM_0011065.3)	5′‐AGCAGGTTCAGGCTAACCAGGAAT‐3′	5′‐AGGTGTCCTGGTTTCGAAGTGTGT‐3′
*mPer2* (NM_011066.3)	5′‐ACCCTGAAAAGGAAGTGCGA‐3′	5′‐GCCATATCTTCTACCGTCTCTAGC‐3′
*mPpar‐α* (NM_011144.6)	5′‐ACGTTTGTGGCTGGTCAAGT‐3′	5′‐TGGAGAGAGGGTGTCTGTGAT‐3′
*mPpar‐γ (*NM_001127330.2)	5′‐TGTGGGGATAAAGCATCAGGC‐3′	5′‐CCGGCAGTTAAGATCACACCTAT‐3′
*mRpl37a* (NM_009084.4)	5′‐CGGCGACATGGCTAAACG‐3′	5′‐ACGGCTCGTCTCTTCATCTTG‐3′
*mScap* (NM_001410582.1)	5′‐TGGGAAGTACAGTGGGGTGA‐3′	5′‐CCACACGCAAGATCGACATG‐3′
*mSlca2a* (NM_031197.2)	5′‐TGTTGGGGCCATCAACATGA‐3′	5′‐GGCGAATTTATCCAGCAGCAC‐3′
*mTnf‐α* (NM_013693.3)	5′‐TTCTCATTCCTGCTTGTGGC‐3′	5′‐ACTTGGTGGTTTGCTACGACG‐3′

### 2.7 Histological Staining

Liver tissue fragments were fixed in 4% paraformaldehyde for 24 h, transferred to 70% ethanol, and then embedded in paraffin. Transverse sections (5 μm) were prepared and mounted on glass slides for hematoxylin and eosin (H&E) staining. The slides were deparaffinized in xylene and hydrated through a gradual ethanol series (absolute alcohol, 96%, and 70%) followed by rinsing in running water. Sections were stained with Harris hematoxylin for 3 min, washed in running water for 2 min, and counterstained with alcoholic eosin for 10 s. Dehydration was then performed through graded ethanol (70%, 96%, and absolute) and xylene baths. Finally, slides were mounted with coverslips using a permanent mounting medium and labeled for morphological analysis. Histological images were obtained using a Leica DM1000 LED microscope at 40 × magnification.

### 2.8 Glycogen Quantification

Glycogen was quantified from 100 mg of liver according to [41]. The samples were boiled in 1 mL of 30% KOH for 1 h, then 100 μL of Na_2_SO_4_ was added, and the mixture was homogenized. After two washes with 70% ethanol, the pellets were resuspended in 1 mL of deionized water at 60°C and diluted in a 1:15 ratio. The absorbance was measured at 650 nm using 2% Antrona solution in concentrated H_2_SO_4_. Glycogen quantification was calculated on the basis of absorbance corrected for tissue mass and normalized by the glucose standard curve, using a microplate reader Epoch (BioTek, Winooski, VT, USA).

### 2.9 Immunoblotting

Protein concentration was determined using the BCA assay (Pierce, Thermo Fisher Scientific, USA). Aliquots containing 15 μg of total protein were mixed with Laemmli sample buffer supplemented with 0.1 M DTT, heated at 96°C for 10 min, and subjected to electrophoresis on a 10% SDS‐PAGE gel for 2 h at 100 V. Proteins were then wet transferred to nitrocellulose membranes (100 V, 1 h), which were subsequently blocked with 5% BSA in TBS‐T (0.1% Tween‐20) for 2 h at room temperature. Membranes were incubated overnight at 4°C with primary antibodies, followed by 1 h of incubation with HRP‐conjugated secondary antibodies at room temperature. Detection was performed using enhanced chemiluminescence (ECL; Thermo Fisher Scientific, USA), and band densitometry was analyzed using ImageJ software (NIH, USA, RRID:SCR_003070). Images were converted to 8‐bit grayscale, and bands corresponding to the proteins of interest were manually selected using the rectangular tool. Band intensity was quantified and normalized either to the housekeeping protein (loading control) or, for phosphorylated proteins, to the respective total protein. Results were expressed in arbitrary units and used for comparisons between experimental groups.

The primary antibodies used were: total AKT (#9272, 1:1000, Cell Signaling, USA, RRID:AB_329827); phospho‐AKT (Ser473) (#4060, 1:2000, Cell Signaling, USA, RRID:AB_2315049); total GSK‐3α/β (ab185141, 1:5000, Abcam, UK, RRID:AB_3718152); phospho‐GSK‐3α/β (Ser21/9) (#9331, 1:1000, Cell Signaling, USA, RRID:AB_329830); PEPCK‐M (ab187145, 1:1000, Abcam, UK, RRID:AB_3718150); FOXO1 (#9454, 1:1000, Cell Signaling, USA, RRID:AB_823503) and phospho‐FOXO1 (Ser256) (#9461, 1:1000, Cell Signaling, USA, RRID:AB_329831); total NF‐κB (#8242, 1:1000, Cell Signaling, USA, RRID:AB_10859369); phospho‐NF‐κB (Ser536) (#3033, 1:1000, Cell Signaling, USA, RRID:AB_331284); β‐actin (A5316, 1:5000, Sigma‐Aldrich, USA, RRID:AB_476743). The secondary antibodies used were anti‐rabbit IgG (#7074, RRID:AB_2099233) and anti‐mouse IgG (#7076, RRID:AB_330924), both from Cell Signaling, USA.

### 2.10 Human Liver Biopsy Samples and Gene Expression Analysis

The study enrolled patients from the Department of Surgery, University Medical Centre Schleswig‐Holstein, Campus Lübeck. All patients with obesity had undergone surgical liver biopsy during the bariatric intervention. The patients were aged between 26 and 62 years and fit the criteria for obesity surgery according to current guidelines, namely, body mass index (BMI) ≥ 40 or ≥ 35 kg/m^2^ and at least one cardiometabolic comorbidity (hypertension, T2DM, dyslipidemia, or OSAS). Pre‐operative evaluation included a detailed medical history, physical examination, and nutritional, metabolic, cardiorespiratory, and psychological assessment. Exclusion criteria were autoimmune, inflammatory, or infectious diseases, viral hepatitis, cancer, or known alcohol consumption (> 20 g/day for women and > 30 g/day for men). Groups were categorized according to liver histology. All patients provided written informed consent. The local ethics committee approved the present clinical investigations. The study was conducted in accordance with the Declaration of Helsinki. Liver samples were stored in PBS on ice for a short period for transport before freezing. Samples were stored at −80°C until further processing. The RNA was extracted using the RNeasy Mini Kit (Qiagen GmbH, Hilden, Germany) according to the manufacturer's instructions. RNA quantity and purity were estimated using the NanoDrop ND‐1000 Spectrophotometer (NanoDrop Technologies, Wilmington, Germany). Complementary DNA (cDNA) was synthesized by reverse transcription using the iScript cDNA Synthesis Kit (Bio‐Rad Laboratories GmbH, Munich, Germany). The gene expression of *GPNMB*, *SREBP*, *FBP1*, and *FOXO1* (Table 1) was quantified by real‐time PCR using the Blue S'Green qPCR Kit (Biozym Scientific GmbH, Hessisch Oldendorf, Germany). Gene expression was normalized to *GAPDH* and quantified as described above.

### 2.11 Statistical Analysis

The power analysis was calculated using the GPower 3.1.9.4 program (RRID:SCR_013726). Statistical analyses were performed using GraphPad Prism version 8.0 (GraphPad Software Inc., San Diego, CA, USA, RRID:SCR_002798) for data from mice and STATA version 14.0 (StataCorp LP, College Station, TX, USA, RRID:SCR_012763) for human data.

For the animal experiments, data normality was assessed using the Shapiro–Wilk test, and the presence of outliers was verified using the ROUT method. The data are presented as a boxplot, showing the median, quartiles, maximum, and minimum values. The statistical tests applied were selected according to the nature of the data and are described in the respective figure legends. Values of *p* < 0.05 were considered statistically significant. For rhythmic analysis, gene expression, RER, and EE data were analyzed using the CircaCompare algorithm [42] to determine rhythmicity, acrophase (phase of the peak in a cosine curve), the Midline Estimating Statistics of Rhythm (MESOR, average level of the rhythm), and the amplitude. The fitted curves were generated using the Cosinor online tool (https://cosinor.online/app/cosinor.php).

For the human data, liver *Gpnmb* expression were considered a dependent variable, whereas diabetes status, diagnosis, age, BMI, medications used, aspartate aminotransferase (AST), ALT, albumin, Fib‐4 index, creatinine, uric acid, TSH, glycated hemoglobin (HbA1c), total cholesterol, LDL, HDL, NAS score, *FOXO1*, *FBP1*, and *SREBP1* were included as explanatory variables. In the bivariate analysis, Pearson's correlation coefficient was used to assess associations between *GPNMB* and normally distributed explanatory variables. In contrast, Spearman's rank correlation was applied for non‐normally distributed variables. The Kolmogorov–Smirnov test was used to evaluate the normality of each variable. Associations between *GPNMB* and categorical variables were examined using Student's *t*‐tests (for comparisons between two groups) or one‐way ANOVA (for comparisons among three or more groups). For the multivariate analysis, a linear regression model was constructed considering *GPNMB* as the outcome variable. Variables with *p* < 0.10 in the bivariate analysis were initially included in the model, and those with *p* < 0.05 were retained in the final model. Potential interactions among explanatory variables were also tested, and when significant interactions were identified, stratified models were estimated to characterize the effect modification better.

## 3 Results

### 3.1 GPNMB Deficiency Leads to Dyslipidemia Without Body Weight Differences After 12 Weeks on HFD + FRUT

**FIGURE 1:**
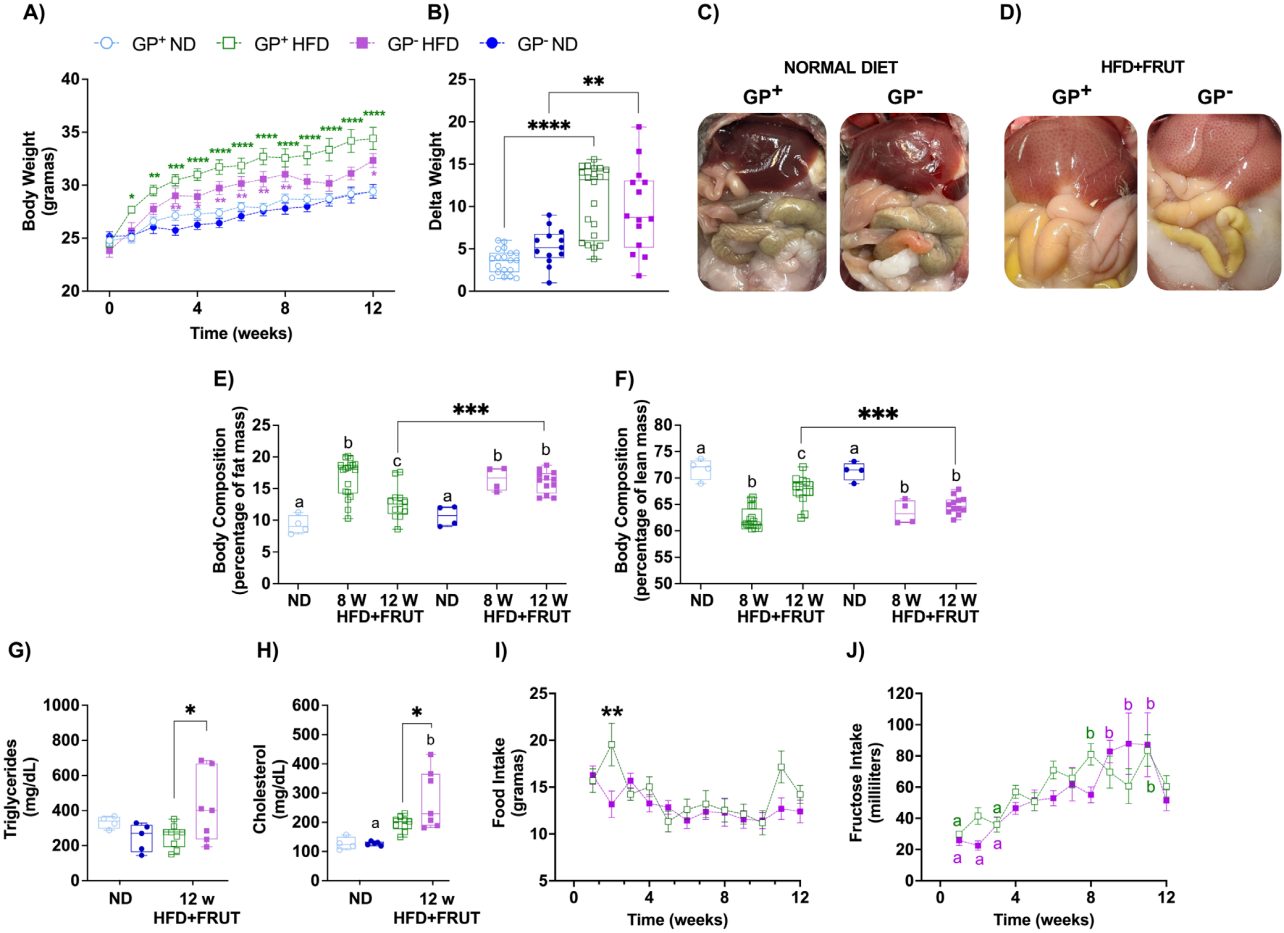
GPNMB deficiency aggravates adiposity and lipid alterations under HFD + FRUT. Body weight of male GPNMB+ and GPNMB‐ mice submitted to a normolipid diet (ND) or high‐fat diet supplemented with fructose (HFD + FRUT) over 12 weeks. Body weight was presented as the weekly average, expressed in grams ± SEM (*n* = 12–20). Asterisks indicate differences between ND and HFD + FRUT within the same genotype. (A) Weight gain expressed in grams ± SEM (*n* = 12–20) (B) Representative photos showing abdominal fat in GPNMB+ and GPNMB‐ mice submitted to ND (C) or HFD + FRUT (D) over 12 weeks. Body composition of GPNMB+ and GPNMB‐ mice after ND or following 8 and 12 weeks of HFD + FRUT (E and F). Body composition data were expressed as percentages ± SEM (*n* = 4–20), showing fat mass (E) and lean mass (F) Plasma levels of triglycerides (G) and cholesterol (H) in GPNMB+ and GPNMB‐ mice fed ND or 12 weeks of HFD + FRUT. Data are presented as milliliters per deciliter (mg/dl) of each animal (*n* = 4–9) in a boxplot, indicating median, quartiles, maximum, and minimum values. Average weekly food (I) and water consumption (J) of GPNMB+ and GPNMB‐ mice fed with HFD + FRUT over 12 weeks. Data are presented as the weekly average of individual consumption, in grams or milliliters ± SEM (*n* = 6–11). The asterisk (*) indicates a significant difference (*p* < 0.05) between mice with functional GPNMB and mutation carriers, calculated by Two‐Way ANOVA followed by the Bonferroni post‐test. Different letters indicate a statistical difference between animals of the same genotype.

To elucidate the role of GPNMB in modulating metabolic responses, we monitored body weight in control (GP^+^) and GPNMB‐deficient (GP^−^) mice fed an HFD + FRUT. Both genotypes showed a significant increase in body weight when fed an HFD + FRUT compared with those fed an ND. In the GP^+^ mice group, weight gain was evident from the first week of HFD + FRUT and persisted throughout the 12 weeks (Figure 1A). In contrast, GP^−^ mice exhibited a delayed onset of weight gain, with a significant increase only emerging from the third week onward. Although no difference in body weight gain was detected between GP^+^ and GP^−^ mice after 12 weeks of HFD + FRUT, both groups gained more weight than ND‐fed mice (Figure 1B). After 12 weeks of HFD + FRUT, both GP^+^ and GP^−^ mice showed increased adiposity compared with ND‐fed controls (Figure 1C,D). In GP^+^ mice, fat mass increased significantly by Week 8, then declined by Week 12, but remained above ND levels. GP^−^ mice exhibited higher fat mass than ND‐fed GP− mice at both 8 and 12 weeks, and significantly higher fat mass than GP^+^ mice at Week 12 under HFD + FRUT (Figure 1E). Under HFD + FRUT conditions, both GP^+^ and GP^−^ mice exhibited reduced lean mass as compared with ND‐fed controls. After 12 weeks of HFD + FRUT, GP^−^ mice showed lower lean mass than GP^+^ mice subjected to the same protocol (Figure 1F). After 12 weeks of diet protocol, GP^−^ mice exhibited higher serum triglyceride and cholesterol levels than GP^+^ mice under HFD + FRUT, whereas no genotype‐dependent differences were detected under ND (Figure 1G,H). To evaluate whether differences in food intake could explain the observed metabolic outcomes, we tracked food and fructose intake throughout the experimental protocol. No significant differences were observed between the genotypes under HFD + FRUT (Figure 1I,J), although both exhibited a gradual increase in fructose intake over time (Figure 1J).

Together, these data indicate that although GPNMB deficiency delays the onset of weight gain, it ultimately leads to greater adiposity, reduced lean mass, and worsened lipid profiles under 12 weeks of HFD + FRUT exposure, independent of food intake.

### 3.2 GPNMB Deficiency Preserves Glycemic Control but Disrupts Energy Expenditure Rhythms After 12 Weeks on HFD + FRUT

**FIGURE 2:**
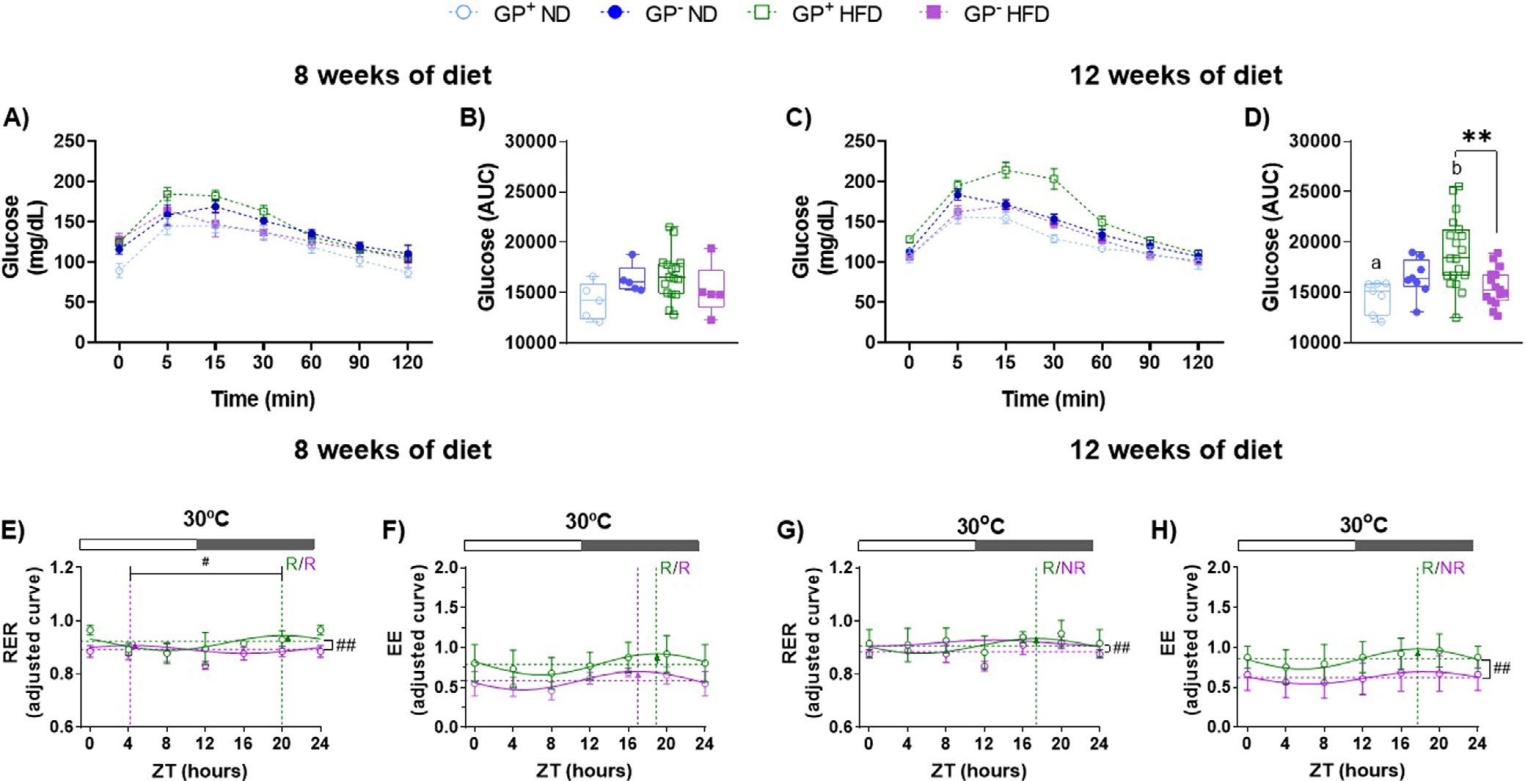
GPNMB deficiency improves glycemic response but compromises circadian rhythms of substrate utilization and energy expenditure in mice fed a HFD + FRUT. Glucose response curve and area under the curve (AUC) of GPNMB+ and GPNMB‐ mice fed with ND or HFD + FRUT for 8 (A and B, respectively) or 12 weeks (C and D, respectively). The values of glycemia are presented as mean ± SEM (*n* = 5–18). The asterisk (*) indicates a significant difference (*p* < 0.05) between mice with functional GPNMB and mutation carriers, calculated by one‐way ANOVA followed by the Bonferroni post‐test. Different letters indicate a statistical difference between animals of the same genotype when comparing ND and HFD + FRUT. Respiratory exchange ratio (RER) and energy expenditure (EE) of GPNMB+ and GPNMB‐ mice fed with HFD + FRUT for 8 (E and F, respectively) or 12 weeks (G and H, respectively). The data are presented as fitted cosine curves (solid lines) generated using the Cosinor Online program for RER and EE values (*n* = 3–4). Data points represent the mean ± SEM. Rhythmic parameters, including acrophase (dotted vertical lines), mesor (dotted horizontal lines), and amplitude (vertical arrows), were estimated using the CircaCompare algorithm. The # symbol denotes a statistically significant difference (*p* < 0.05) in rhythmic parameters between groups with functional GPNMB and GPNMB mutation carriers, as determined by CircaCompare. The white and gray rectangular shapes above the graph indicate the time points in the light (ZT0–ZT12) and dark (ZT12–ZT24) phases, respectively. ZT (zeitgeber time). *R* (rhythmic). NR (non‐rhythmic).

We investigated the role of GPNMB on blood glucose levels during the ipGTT in GP^+^ and GP^−^ mice fed with ND or HFD + FRUT. Both genotypes showed similar glucose responses after 8 weeks of HFD + FRUT (Figure 2A,B). After 12 weeks of HFD + FRUT, GP^+^ mice showed impaired glucose metabolism, as evidenced by a delayed clearance of plasma glucose compared to ND. Surprisingly, GP^−^ mice fed with HFD + FRUT showed no impairment in glycemic control, showing a faster glucose clearance compared to GP^+^ mice on the same diet protocol. This response is not attributed to diet, but rather to the absence of GPNMB itself (Figure 2C,D). Indirect calorimetry analyses under thermoneutral conditions (30°C) indicated that both genotypes predominantly used carbohydrates as an energy source (RER values close to 1). After 8 and 12 weeks of dietary protocol, no significant differences in substrate utilization between the genotypes were observed (Figure 2E,G). At 8 weeks on HFD + FRUT, rhythm analyses revealed that both genotypes maintained the diurnal rhythmicity of RER. However, GP^−^ mice exhibited a significantly reduced MESOR of RER and a phase shift (e.g., 12 h) from the dark to light phase (Figure 2E and Table S1). Conversely, a diurnal rhythm in EE was observed in both genotypes, but GP^−^ mice exhibited a lower MESOR compared to GP^+^ animals without any changes in rhythmic parameters (Figure 2F and Table S1). After 12 weeks of dietary protocol, GP^−^ mice lost diurnal rhythmicity in both RER and EE, exhibiting reduced EE amplitude, and reduced MESOR levels in both parameters (Figure 2G,H and Table S1).

These findings show that loss of GPNMB uncouples glycemic control from circadian regulation of energy expenditure, with GP^−^ mice maintaining glucose tolerance but showing disrupted EE rhythms 12 weeks after HFD + FRUT.

### 3.3 GPNMB Deficiency Alters Hepatic Diurnal and Metabolic Gene Expression in Response to HFD + FRUT

**FIGURE 3:**
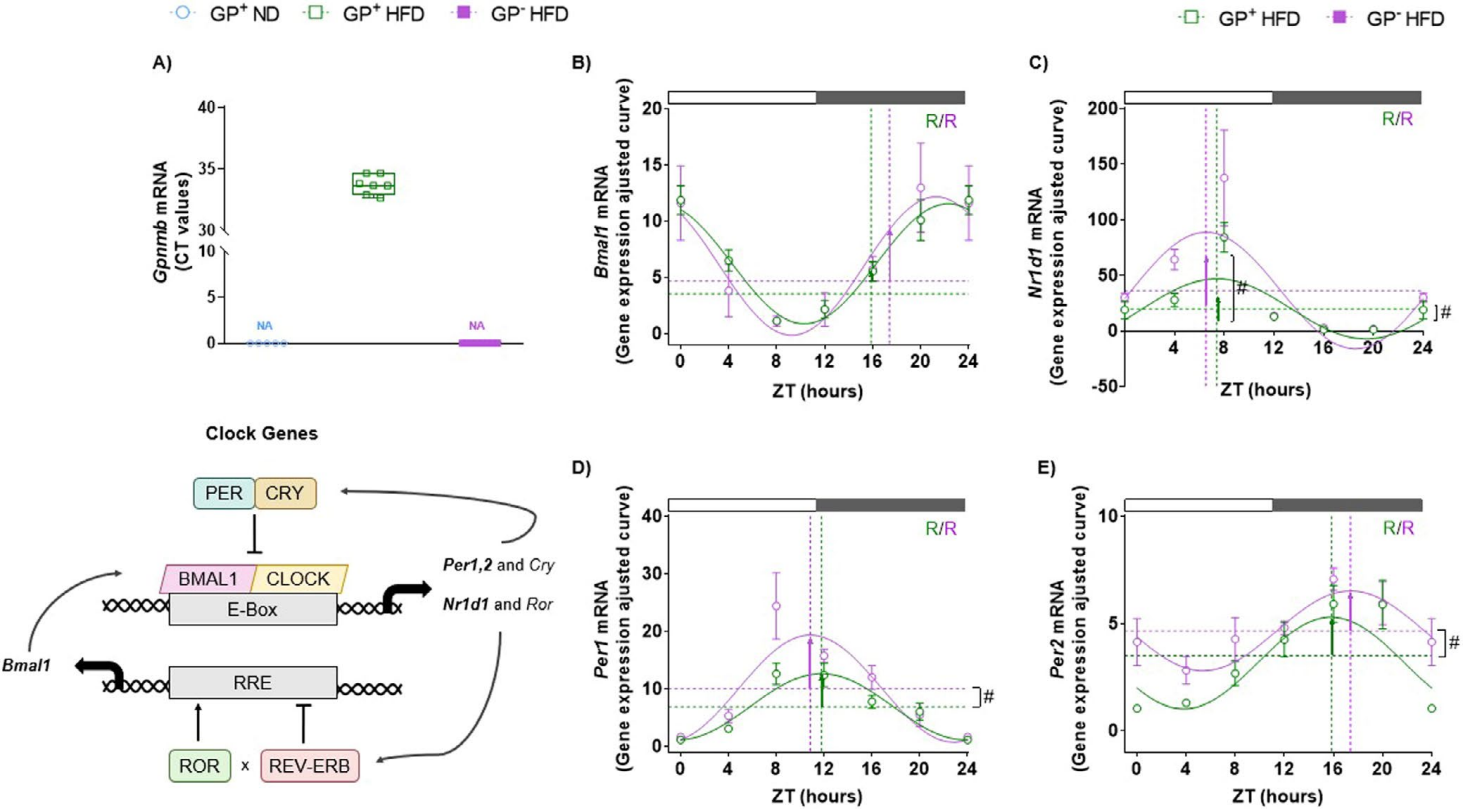
GPNMB deficiency alters the rhythmic parameters of clock genes in the liver of mice subjected to HFD + FRUT. Gene expression of *Gpnmb* in the liver of GPNMB+ mice fed with ND, GPNMB+ and GPNMB‐ mice fed with 12 weeks of HFD + FRUT. The data were presented in boxplots showing the median, quartiles, maximum, and minimum relative of expression levels (CT) (*n* = 5–8) (A). Rhythmic profile of Bmal1, Nr1d1, Per1, and Per2 gene expression in the liver of GPNMB+ and GPNMB‐ mice fed with 12 weeks of HFD + FRUT (B–E). The data are presented as fitted cosine curves (solid lines) generated using the Cosinor Online program. Data points represent the mean ± SEM relative to the lowest mean of the GPNMB+ HFD + FRUT group (*n* = 3–8). Rhythmic parameters, including acrophase (dotted vertical lines), mesor (dotted horizontal lines), and amplitude (vertical arrows), were estimated using the CircaCompare algorithm. The # symbol denotes a statistically significant difference (*p* < 0.05) in rhythmic parameters between groups with functional GPNMB and GPNMB mutation carriers, as determined by CircaCompare (B–E). The white and gray rectangular shapes above the graph indicate the time points in the light (ZT0–ZT12) and dark (ZT12–ZT24) phases, respectively. ZT (zeitgeber time). *R* (rhythmic).

Given the systemic metabolic alterations observed, we focused on the liver as a key site for GPNMB‐dependent regulation in mice fed with HFD + FRUT for 12 weeks. Specifically, we investigated the hepatic circadian clock, a major regulator of glucose homeostasis and energy metabolism [13]. We confirmed that *Gpnmb* expression is markedly induced in GP^+^ control mice fed HFD + FRUT (Figure 3A). This induction was observed exclusively in the GP^+^ group, as the premature termination of *Gpnmb* in GP^−^ mice impairs gene expression, preventing its detection. These findings support GPNMB involvement in the hepatic response to HFD + FRUT. Diurnal clock gene profiles of *Bmal1*, *Per1*, *Per2*, and *Nr1d1* were rhythmic in both genotypes (Figure 3B–E). Although we found no alterations in rhythmic parameters of *Bmal1*, GP^−^ mice showed increased amplitude and a higher MESOR (*p* = 0.0568) of *Nr1d1* (Figure 3C and Table S2). Both *Per1* and *Per2* genes displayed increased MESOR in GP^−^ mice compared to GP^+^ (Figure 3D,E and Table S2). A tendency of increased amplitude was observed for *Per2* in the liver of GP^−^ mice (*p* = 0.06).

**FIGURE 4:**
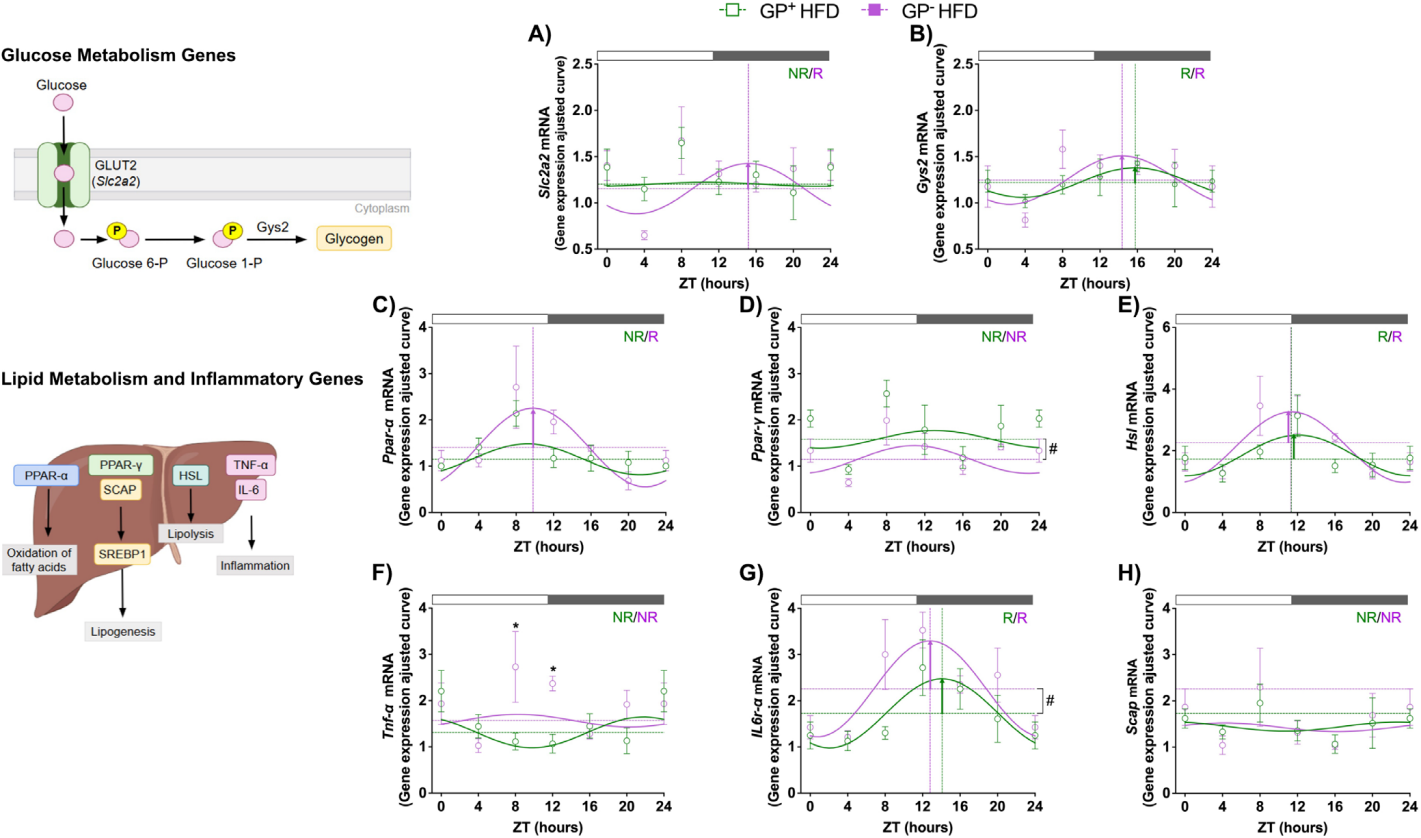
The absence of GPNMB modulates the temporal expression of metabolic genes and inflammatory markers, contributing to changes in glycemic, lipid, and systemic inflammatory profiles under HFD + FRUT. Rhythmic profile of Slc2a2, Gys2, Ppar‐α, Ppar‐γ, Hsl, Scap, Tnf‐α, and Il6r‐α gene expression in the liver of GPNMB+ and GPNMB‐ mice fed with 12 weeks of HFD + FRUT (A–H). The data are presented as fitted cosine curves (solid lines) generated using the Cosinor Online program. Data points represent the mean ± SEM relative to the lowest mean of the GPNMB+ HFD + FRUT group (*n* = 3–8). Rhythmic parameters, including acrophase (dotted vertical lines), mesor (dotted horizontal lines), and amplitude (vertical arrows), were estimated using the CircaCompare algorithm. The # symbol denotes a statistically significant difference (*p* < 0.05) in rhythmic parameters between groups with functional GPNMB and GPNMB mutation carriers, as determined by CircaCompare (A–H). The white and gray rectangular shapes above the graph indicate the time points in the light (ZT0–ZT12) and dark (ZT12–ZT24) phases, respectively. ZT (zeitgeber time). *R* (rhythmic). NR (non‐rhythmic).

Considering the changes in the glycemic profile and the increases in fat mass, triglycerides, and cholesterol levels, we evaluated the temporal expression of elements involved in glucose (Figures 4A,B and Table S2) and lipid metabolism (Figures 4C–F and Table S2) in mice after 12 weeks of HFD + FRUT. Rhythmic analysis of *Slc2a2*, which encodes the GLUT2 transporter, revealed a diurnal oscillatory pattern only in GP^−^ mice, with no significant differences in rhythmic parameters (Figure 4A). In contrast, the expression of *Gys2*, encoding glycogen synthase 2, showed a rhythmic profile in both groups without significant changes in rhythmic parameters (Figure 4B). *Ppar‐α* expression was rhythmic only in GP^−^ mice, but without alteration in any rhythmic parameter (Figure 4C). However, *Ppar‐γ* was arrhythmic in both groups, with GP^−^ mice showing a significantly lower MESOR than GP^+^ mice (Figure 4D). The expression of *Hsl*, which encodes hormone‐sensitive lipase, showed a diurnal oscillation in both genotypes without changes in rhythmicity parameters (Figure 4E), whereas *Scap* expression was arrhythmic in both groups (Figure 4F). We also evaluated the expression of key pro‐inflammatory cytokines (Figure 4G,H). Rhythmic analysis did not reveal a temporal pattern in *Tnf‐α* expression in either group (Figure 4G). In contrast, *Il6r‐α* expression exhibited a diurnal rhythm in both genotypes, with a significantly higher MESOR in GP^−^ mice (Figure 4H). Although the amplitude of *Il6r‐α* was not altered in GP^−^ mice, the expression of this gene was increased at ZT8 (*p* = 0.0415).

Overall, we show that GP‐mice have increased expression of clock genes (e.g., *Per1*, *Per2*, *Nr1d1*) as well as some inflammatory genes (*Tnf‐α* and *Il6r‐α*). These findings suggest they may increase circadian clock output and gene expression of inflammatory genes in the liver under HFD + FRUT conditions.

### 3.4 Loss of GPNMB Disrupts Time‐Dependent Hepatic Insulin Signaling and Promotes Gluconeogenic and Inflammatory Adaptations

**FIGURE 5:**
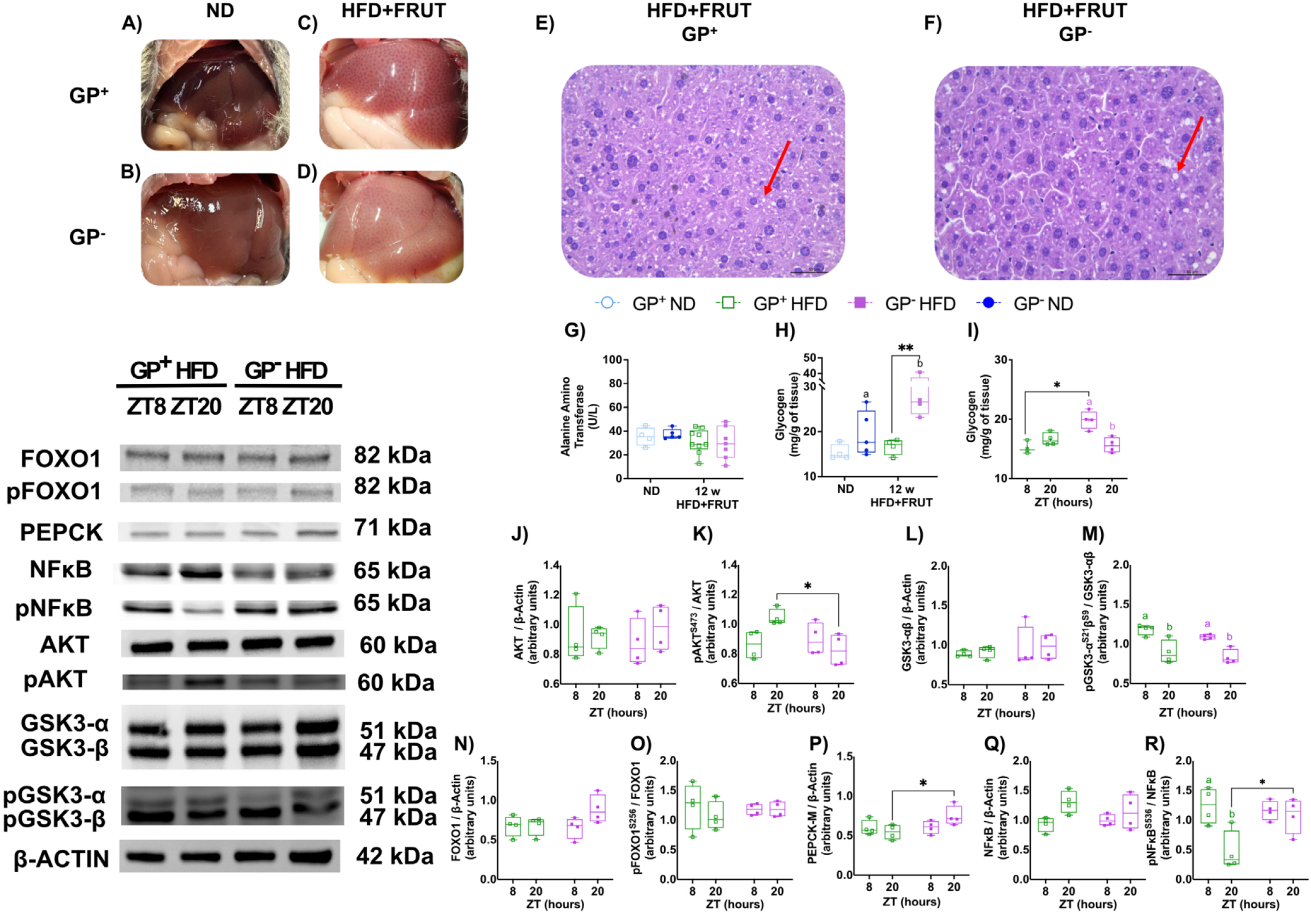
GPNMB deficiency alters hepatic glycogen dynamics, gluconeogenic pathways, and inflammatory adaptations in mice fed a HFD + FRUT, without inducing acute liver injury. Representative liver tissue image from GPNMB+ and GPNMB‐ mice fed ND (A and B) or HFD + FRUT for 12 weeks (C and D). H&E staining of liver tissue from GPNMB+ and GPNMB‐ mice fed 12 weeks of HFD + FRUT (E and F). Plasma alanine aminotransferase levels in GPNMB+ and GPNMB‐ mice fed ND or 12 weeks of HFD + FRUT (G). Data are presented as units per liter (U/L) for each animal (*n* = 4–9). Hepatic glycogen concentration in GPNMB+ and GPNMB‐ mice fed ND or 12 weeks of HFD + FRUT (H) and GPNMB+ and GPNMB‐ mice fed for 12 weeks of HFD + FRUT at ZT8 (15 h) and ZT20 (23 h) (I). Data are presented as micrograms per gram (mg/g) for each animal (*n* = 4–5). Protein expression of AKT, pAKT, GSK3‐α and β, pGSK3‐α and β, FOXO1, pFOXO1, PEPCK‐M, NF‐κB, and pNF‐κB normalized by β‐Actin in the liver tissue of mice of both genotypes fed for 12 weeks of HFD + FRUT at ZT8 and ZT20 (*n* = 4 per time point) (J–R). Data are presented in a boxplot, indicating median, quartiles, maximum, and minimum values. The asterisk (*) indicates a significant difference (*p* < 0.05) between mice with functional GPNMB and mutation carriers, calculated by Two‐Way ANOVA, followed by the Bonferroni post‐test. Different letters indicate a statistical difference between animals of the same genotype.

Liver macroscopy showed that livers of both genotypes were paler in color, irrespective of genotype, compared to their ND controls (Figures 5A–D), and this was confirmed by the absence of marked microscopic histological alterations, despite lipid droplet accumulation (Figure 5E,F). In line with these findings, ALT levels remained unchanged, indicating the absence of acute hepatic injury under these conditions (Figure 5G), suggesting simple steatosis.

Given the observed alterations in glycemic control and diurnal energy expenditure in GP^−^ mice, hepatic glycogen quantification provides a critical functional readout of glucose storage and utilization in the liver. Since GPNMB deficiency preserved glycemic control under HFD + FRUT, despite increased adiposity and disrupted energy expenditure rhythms, we hypothesized that liver glycogen dynamics could contribute to the observed metabolic phenotype. Quantification of hepatic glycogen at ZT4 in mice fed with ND or HFD + FRUT showed higher glycogen levels in GP^−^ mice under HFD + FRUT compared to both GP^+^ mice on the same diet and GP^−^ mice fed an ND (Figure 5H). To assess diurnal glycogen storage, additional measurements were taken at ZT8 (late rest phase) and ZT20 (active/feeding phase) in mice fed with HFD + FRUT. At ZT8, GP^−^ mice had higher glycogen levels compared to GP^+^ mice, reinforcing genotype‐specific differences during the fasting phase. At ZT20, glycogen levels in GP^−^ mice were lower than at ZT8. However, no significant differences between genotypes were observed at ZT20 (Figure 5I).

Next, we investigated the insulin signaling pathway to better understand hepatic glucose homeostasis. We quantified key proteins involved in insulin signaling. Specifically, we assessed levels of AKT and GSK3‐α/β. Total AKT levels did not differ significantly between GP^+^ and GP^−^ mice fed HFD + FRUT at either ZT8 or ZT20 (Figure 5J). However, a notable reduction in AKT phosphorylation at serine 473, required for AKT activation, was observed in GP^−^ mice at ZT20 compared to GP^+^ mice at the same time point, indicating a time‐specific reduction in AKT activation in GP^−^ mice. No significant temporal variation in AKT phosphorylation was detected between ZT8 and ZT20 within either genotype (Figure 5K). Similarly, total GSK3‐α/β expression remained unchanged between genotypes and time points (Figure 5L). However, phosphorylation of GSK3‐α (Ser21) and GSK3‐β (Ser9), inhibitory sites that regulate glycogen synthase, was reduced at ZT20 compared to ZT8 in both GP^+^ and GP^−^ mice (Figure 5M), indicating a diurnal regulation of GSK3 activity that could influence glycogen synthesis capacity during the feeding period.

Considering that AKT phosphorylation was reduced, whereas GSK3‐β was unaffected, we next evaluated FOXO1, a transcription factor that regulates genes involved in gluconeogenesis, such as *Pck1/Pck2 and* G6Pase [43]. Total FOXO1 levels did not differ between the GP^+^ and GP^−^ mice at either ZT8 or ZT20 (Figure 5N). Phosphorylation of FOXO1 at Ser256 promotes its translocation from the nucleus to the cytoplasm, resulting in the inactivation of the transcription factor and reducing the expression of gluconeogenic genes [44]. Although no significant difference in FOXO1 phosphorylation was detected between groups (Figure 5O), a trend was observed (two‐way ANOVA, F(1,12) = 4.393, *p* = 0.0580). As the time factor was not significant (F(1,12) = 0.0029, *p* = 0.9580), data from ZT8 and ZT20 were combined for analysis, and an unpaired *t*‐test revealed a significant reduction in FOXO1 phosphorylation in GP^−^ mice compared to GP^+^ mice (*p* = 0.0401). To further explore potential mechanisms underlying altered hepatic glucose metabolism, we measured PEPCK‐M (the mitochondrial isoform of phosphoenolpyruvate carboxykinase), which plays a crucial role in regulating gluconeogenic pathways, converting oxaloacetate to phosphoenolpyruvate inside the mitochondria [12, 45]. Notably, PEPCK‐M expression was significantly increased at ZT20 only in GP^−^ mice subjected to HFD + FRUT, compared to GP^+^ mice under the same conditions (Figure 5N), indicating a genotype‐specific enhancement in gluconeogenic signaling during the active phase.

Because hepatic insulin signaling and glucose handling are tightly interconnected with inflammatory pathways in a time‐dependent manner [46, 47, 48], we next examined NF‐κB signaling as a potential integrative mechanism linking metabolic and circadian alterations in the absence of GPNMB. Total NF‐κB protein levels did not differ between GP^+^ and GP^−^ mice fed an HFD or between ZTs (Figure 5Q). In contrast, phosphorylation of NF‐κB at Ser536 (a site that enhances transcriptional activity) was significantly reduced in the liver of GP^+^ mice at ZT20, both compared to GP^−^ mice at the same time point and to GP^+^ mice at ZT8 (Figure 5R). Together, these findings indicate that the presence of functional GPNMB is associated with reduced activation of the NF‐κB pathway during the active/feeding phase.

Overall, these findings reveal that GPNMB deficiency is associated with altered hepatic glycogen storage, impaired insulin signaling during the feeding phase, enhanced gluconeogenic signaling, and a pro‐inflammatory hepatic profile marked by increased NF‐κB activation. Together, these alterations may contribute to the paradoxical preservation of glycemia despite systemic metabolic disruption.

### 3.5 MASLD Is Correlated With *GPNMB* Expression and Influenced by Diabetes

We investigated the translational relevance of our findings in MASLD patients. Liver samples from healthy obese individuals (HO), patients with simple steatosis (MASL), and patients with MASH were collected, along with clinical data. This approach enabled evaluation of the relationship between *GPNMB* gene expression and metabolic markers indicative of liver damage severity.

We found that GPNMB expression is higher in patients with MASH compared to MASL and HO individuals. Interestingly, MASH patients with diabetes who all received.

**FIGURE 6:**
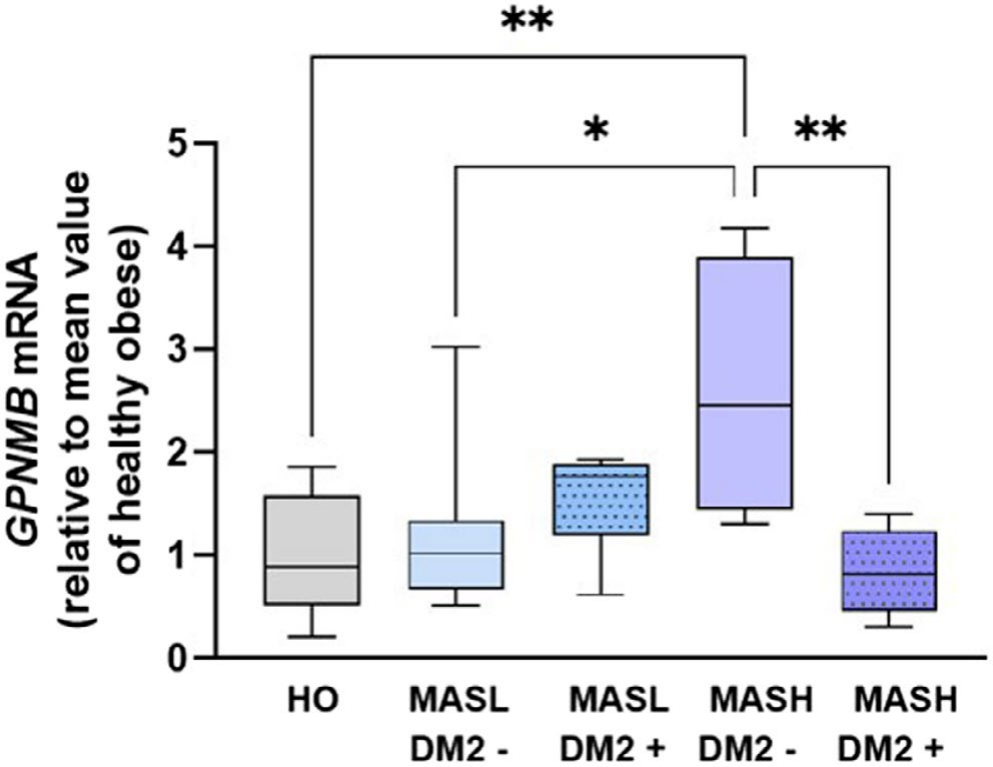
Increase in hepatic expression of GPNMB is associated with the severity of metabolic steatohepatitis. Gene expression of GPNMB in the livers of healthy obese (HO), moderate hepatic steatosis (MASL), and metabolic steatohepatitis (MASH) patients, with or without type 2 diabetes mellitus (DM2). Gene expression was normalized to the mean HO group value (*n* = 4–10). Data are presented as boxplots representing the mean ± interquartile range. Significant differences between groups were determined by one‐way ANOVA followed by Tukey's post hoc test, with asterisks (*) indicating *p* < 0.05.

Treatment (mostly metformin ± GLP1‐RA and/or SGLT‐2 inhibitors) showed a reduction in GPNMB expression, similar to levels seen in MASL patients (Figure 6).

Bivariate analyses were performed to evaluate the correlations between liver *GPNMB* expression and clinical parameters and other metabolic relevant genes (*SREBP1*, *FOXO1*, and *FBP1*, Table S3). When these correlations were incorporated into the linear regression model, diabetes, liver diagnoses [on the basis of the NAFLD Activity Score (NAS), which defines HO, MASL, and MASH conditions], and glycated hemoglobin were the independent variables that explained the changes in GPNMB expression (Table 2, *R* ^2^ = 74.5%). When the model was stratified by diabetes status, the diagnosis of HO, MASL, and MASH explained alterations in GPNMB expression in patients without diabetes (*R* ^2^ = 82.4%). In contrast, only *FOXO1* expression explained these alterations in patients without diabetes (*R* ^2^ = 43.3%) (Table 2). This might be due to the pharmacological treatments which patients with diabetes underwent. Specifically, nine patients received metformin, five GLP1‐RA, one SGLT‐2 inhibitor, and three insulin; only 2 out of 11 patients with diabetes were not under pharmacological treatment for diabetes. The bivariate analyses did not find any correlations between pharmacological treatments and *GPNMB* expression, except for a tendency for metformin treatment (*p* = 0.14, Table S4).

**TABLE 2:** Linear regression model considering GPNMB as outcome.

	Coefficient	Standard error	*p*	95% CI coefficient
**Initial model (*R* ^2^ = 74.5%)**				
Diabetes				
No	Ref.			
Yes	1.61	0.69	0.028	[0.19; 3.03]
Diagnosis				
HO	Ref.			
MASL	2.99	0.38	< 0.001	[2.21; 3.77]
MASH	2.42	0.5	< 0.001	[1.38; 3.45]
Glycated hemoglobin	–0.36	0.16	0.036	[–0.69; –0.03]
*FOXO1* mRNA	0.21	0.13	0.121	[–0.06; 0.49]
Diabetes × Diagnosis Interaction				
1	4.31	1.12	0.001	[2.00; 6.62]
2	–1.83	0.75	0.021	[–3.37; –0.30]
**Model for group without diabetes (*R* ^2^ = 82.4%)**				
Diagnosis				
HO	Ref.			
MASL	2.96	0.3	< 0.001	[2.33; 3.58]
MASH	2.34	0.41	< 0.001	[1.48; 3.19]
Glycated hemoglobin	−0.11	0.48	0.816	[−1.11; 0.88]
*FOXO1* mRNA	0.11	1.76	0.094	[−0.04; 0.41]
**Diabetes group model (*R* ^2^ = 43.3%)**				
Diagnosis				
MASL	Ref.			
MASH	0.98	0.82	0.269	[−0.95; 2.91]
Glycated hemoglobin	−0.45	0.25	0.117	[−1.06; 0.15]
*FOXO1* mRNA	1.04	0.37	0.028	[0.15; 1.92]

Taken altogether, our findings demonstrate that *GPNMB* expression increases with MASLD progression and decreases in MASH patients with diabetes undergoing treatment.

## 4 Discussion

In this study, we demonstrate that the GPNMB protein plays an important role in regulating energy homeostasis and modulating hepatic gluconeogenesis in a time‐dependent manner. By employing an animal model with a nonfunctional GPNMB protein, we showed that GPNMB deficiency is associated with body fat accumulation and dysregulation of hepatic insulin signaling. Paradoxically, GPNMB deficiency caused a decoupling of glucose and lipid homeostasis, as GP^−^ mice showed improved glycemic response even in the face of worsening adiposity and loss of circadian rhythms in energy expenditure.

GPNMB is upregulated in response to HFD‐induced liver damage in mice and humans [8, 10, 34, 35, 49, 50, 51, 52, 53]. GPNMB expression is positively correlated with obesity [54] and the progression of liver disease [54, 55]. Higher GPNMB has been associated with inflammatory processes, with adipocytes and resident macrophages being the main source of this protein ([30] and Figure S1 A–B). Although a positive correlation has been found between GPNMB and inflammation, this protein's role is in immune‐regulatory processes. GPNMB deficiency increased macrophage infiltration in WAT, with a predominance of CD11c^+^ proinflammatory M1‐like macrophages. Accordingly, the expression of inflammatory cytokines (TNF‐α, IL1β, and IL‐6) was significantly elevated in both WAT and liver, and hepatosteatosis was aggravated in GPNMB‐KO mice [30]. Our data reinforce this response, since the absence of functional GPNMB is associated with enhanced hepatic inflammatory signaling, as evidenced by increased activation of the NF‐κB pathway and hepatic *Tnf‐α* expression (Figures 4F and 5R). These findings suggest that GPNMB limits hepatic inflammatory signaling in a time‐dependent manner, acting as a negative modulator of inflammation under obesogenic conditions. In obesity, serum GPNMB levels increase as it is cleaved into its soluble form (sGPNMB) by adipocytes [30] and may act in other tissues, such as the liver [55, 56].

In response to a metabolic challenge, like HFD, GPNMB expression increases in the WAT [30] and in the liver (Figure 3A,B). Thus, GPNMB expression increases markedly in pathological contexts such as T2DM and fatty liver diseases, including MASLD and MASH [28, 30, 54, 55], supporting its protective role against obesity‐associated inflammation by limiting macrophage infiltration and activation in metabolic tissues [30]. Analysis of liver transcriptomes from public databases [8, 33, 34, 35, 57] showed that *Gpnmb* expression increases in steatohepatitis, whereas such an increase is not observed in models of simple steatosis (Figure S2 A–E). Importantly, Eckel Mahan et al.'s protocol involved 10 weeks of HFD, similar to ours, and no difference in *Gpnmb* was observed. Despite the similarities between our protocol and Eckel Mahan's protocol, a key difference is the fructose intake in the drinking water, which is only present in our protocol. Importantly, our experimental model is predominantly characterized by simple hepatic steatosis (Figure 5E,F), as the data do not indicate a robust inflammatory phenotype. This is supported by the absence of histological changes in the liver (Figure 5A–F) and unaltered serum ALT levels (Figure 5G). Therefore, GPNMB emerges as a potential modulator of metabolic adaptations to caloric excess and high fructose consumption and could also be used as a key target for the progression of MASLD in murine liver.

Although body weight gain was similar between groups after 12 weeks of HFD + FRUT (Figure 1A,B), the absence of GPNMB resulted in significantly more pronounced fat deposition (Figure 1E), and elevations in plasma triglyceride and cholesterol levels (Figure 1G,H), independently of changes in food intake (Figure 1I,J). Consistent with this, we observed a reduction in hepatic pAKT and pFOXO1 levels in GP‐mice (Figure 5K,O), indicating impaired insulin signaling and altered downstream signaling in gluconeogenic pathways. This disruption in AKT–FOXO1 signaling further supports the notion of compromised hepatic metabolic adaptation in GPNMB‐deficient mice. These data reinforce that the metabolic changes observed result from intrinsic mechanisms of energy storage and mobilization.

Although the absence of GPNMB causes significant changes in lipid metabolism, glucose control remains preserved, as evidenced by faster glucose clearance in GP^−^ mice (Figure 2C,D). Consistent with our data, mice treated with anti‐GPNMB antibodies showed faster glucose clearance than control mice in response to diet‐induced obesity, because of increased glucose uptake in the liver and brown adipose tissue [54]. Conversely, overexpression of GPNMB in hepatocytes from mice fed a HFD induces slower plasma glucose clearance, leading to glucose intolerance and body fat accumulation [54]. Our findings reveal an apparent decoupling between systemic metabolic health and glucose homeostasis. The maintenance of glucose tolerance, even in the context of exacerbated adiposity and lipid dysfunction, suggests that compensatory hepatic mechanisms could sustain normoglycemia. Unlike the previous study [54], our findings are based on a global knockout mouse model, which prevents direct comparison. In our experimental model, the preservation of diurnal rhythmicity in *Slc2a2* expression is particularly notable, observed exclusively in GP^−^ mice (Figure 4A and Table S2), following a pattern similar to that described in WT mice fed a normolipidic diet [58]. In addition, we found that under thermoneutral conditions, both genotypes presented RER values close to 1.0 (Figure 2E,G). This indicates a predominant reliance on carbohydrates as the primary energy source [59, 60]. Furthermore, the absence of GPNMB resulted in significantly elevated levels of hepatic glycogen after the HFD + FRUT protocol (Figure 5H), especially during the resting/fasting phase (Figure 5I), suggesting a greater capacity for glucose storage during feeding and more efficient mobilization during fasting. On the other hand, in the presence of GPNMB, glycogen levels remained stable throughout the light–dark cycle, possibly reflecting slower glucose metabolism, or the expected time‐of‐day changes [58] were not captured by our two sampling times. These data and our findings indicate that GPNMB not only influences carbohydrate metabolism through its action on hepatic glucose uptake but also acts as a modulator of the temporal control of glycemic metabolism.

Insulin‐induced hepatic signaling stimulates glycogen synthesis by activating AKT, whose phosphorylation at serine 473 inhibits the GSK3‐α/β isoforms. Inhibition occurs by phosphorylation of N‐terminal residues (Ser21 in GSK3‐α and Ser9 in GSK3‐β), releasing glycogen synthase (GS) activity [43, 61, 62]. In the absence of GPNMB, a reduction in AKT phosphorylation was observed only at the active phase compared to GP^+^ mice (Figure 5K). However, this decrease in AKT activation was not reflected in lower inhibitory phosphorylation of GSK3‐α/β in the corresponding residues when compared to the GP^+^ group. What was identified was a reduction in inhibitory phosphorylation of GSK3‐α/β specifically during the active phase in both genotypes (Figure 5M), suggesting that this modulation is time‐dependent (active‐rest cycle phase) and occurs independently of GPNMB functionality. Thus, this pattern suggests that the classical glycogen synthesis pathway remains functionally unaltered. In line with this, adipocytes exposed to conditioned medium from macrophages lacking GPNMB also showed reduced pAKT levels [30]. Despite reduced AKT phosphorylation in GP^−^ mice, the absence of changes in the inhibitory phosphorylation of GSK3 further supports the idea that this pathway is not the main contributor to glycogen accumulation during the activity/feeding phase. It is plausible to speculate that other targets regulated by AKT are contributing to glycogen regulation. Among them, the transcription factor FOXO1 is of relevance, whose activity is controlled by AKT‐mediated phosphorylation and regulates the expression of key genes of gluconeogenesis, such as *Pck1* and *Pck2* (PEPCK) and *G6pc* (G6Pase) [63]. The reduction in hepatic AKT phosphorylation in GP^−^ mice led to decreased FOXO1 phosphorylation at Ser256. This promotes increased retention of FOXO1 in the nucleus, thereby enhancing its activity as a transcription factor. Consequently, there is an increase in the expression of gluconeogenic genes, including *Pck2*, which is one of the primary targets of FOXO1, promoting greater hepatic gluconeogenesis [43, 44]. Thus, we concluded that a reduction in AKT signaling in GP^−^ directly contributes to the nuclear activation of FOXO1 and the subsequent induction of PEPCK‐M (Figure 6).

This mechanism, therefore, offers an alternative explanation for the persistent accumulation of glycogen observed in the absence of GPNMB, regardless of GSK3 regulation. Additionally, the absence of GPNMB led to increased hepatic PEPCK‐M expression, a marker of increased gluconeogenic activity, which may contribute to maintaining blood glucose levels through non‐glycemic molecular mechanisms, even in the face of peripheral insulin resistance. The increase in glycogen levels during the resting/fasting phase (Figure 5I) suggests a lower mobilization of hepatic reserves, possibly associated with this increase in PEPCK‐M. This metabolic adaptation may indicate a glycogen preservation strategy, with greater dependence on gluconeogenesis rather than glycogenolysis to sustain blood glucose levels during fasting. Together, our findings indicate that functional GPNMB deficiency triggers specific temporal adaptations in hepatic glucose metabolism that allow glycemic control to be maintained despite a metabolically adverse systemic environment.

Beyond canonical insulin signaling, hepatic glucose metabolism is regulated by inflammatory signaling pathways. NF‐κB activation in the liver has been shown to impair insulin signaling by interfering with AKT phosphorylation, while promoting gluconeogenic programs through transcriptional and post‐transcriptional mechanisms [64, 65, 66]. In hepatocytes, activation of NF‐κB increases the expression of proinflammatory cytokines such as TNF‐α, which directly suppresses insulin receptor signaling and favors FOXO1 nuclear activity, thereby enhancing PEPCK and G6Pase expression [67, 68]. Consistent with this, our data demonstrate that the absence of functional GPNMB is associated with increased hepatic NF‐κB activation, reduced AKT phosphorylation, decreased FOXO1 inhibitory phosphorylation, and increased PEPCK‐M expression in a time‐dependent way (Figure 5J–R). This temporal alignment supports the interpretation that increased NF‐κB signaling contributes to alterations in hepatic glucose metabolism in GP^−^ mice, characterized by enhanced gluconeogenic signaling and altered glycogen handling, rather than canonical insulin‐driven glycogen synthesis. Importantly, these findings strengthen the concept that GPNMB acts as a negative modulator of inflammatory signaling in the liver, thereby limiting NF‐κB‐dependent metabolic disruption under diet‐induced metabolic stress.

Circadian regulation of hepatic metabolism is crucial for energy homeostasis, since the liver controls well‐established circadian functions such as carbohydrate, lipid, and protein metabolism [7, 8, 69]. We demonstrated that the *Bmal1*, *Nr1d1*, *Per1*, and *Per2* genes, key elements of the temporal regulatory loop, show preserved diurnal rhythmicity independent of GPNMB functionality (Figure 3C–F and Table S1). However, we observed that the *Nr1d1* and *Pers* genes showed increased MESOR in the absence of GPNMB, with increased expression occurring at specific times during the light phase: *Per2* at the beginning of the dark phase, whereas *Per1* and *Nr1d1* were increased in the transition to the dark phase (Figure 3D–F and Table S1). It should also be noted that, in the case of *Nr1d1*, the increase in MESOR was accompanied by a significant increase in amplitude (Figure 3D and Table S1). These data suggest that the absence of GPNMB affects the regulation of molecular clock components. The anti‐phase relationship between the *Pers/Bmal1* genes, a fundamental characteristic of a preserved rhythmic profile [70], was maintained in our model. In mice with MASH, a phase advance of approximately 4 h on average is observed in the rhythmic profile of clock genes [8]. Thus, our results highlight the resilience of the temporal regulation mechanism in the liver, as changes in the liver clock modulate rhythm parameters without compromising the oscillatory profile in the HFD + FRUT protocol. Importantly, alterations in rhythmic parameters were sufficient to affect downstream processes, such as AKT phosphorylation and glucose storage, in a time‐dependent manner. REV‐ERBα acts as a critical transcriptional repressor linking the circadian clock to hepatic lipid and glucose metabolism [71], suppressing genes involved in lipogenesis and inflammation. The increased amplitude and mean levels of *Nr1d1* observed here may represent an adaptive response to the metabolic stress induced by GPNMB deficiency. Although *Gpnmb* deficiency is associated with alterations in hepatic clock gene expression and systemic metabolic rhythms, the present study does not allow discrimination between primary effects on central circadian regulation and secondary changes driven by metabolic or inflammatory dysregulation. Importantly, inflammatory and circadian systems are linked through a bidirectional crosstalk. NF‐κB signaling can directly interfere with the molecular clock, as the p65 subunit interacts with BMAL1 and represses BMAL1/CLOCK‐driven transcription, leading to alterations in rhythmic parameters such as amplitude [46]. Conversely, core clock components, including CRY and PER proteins, modulate NF‐κB activity [46]. Together, these interactions support the notion that disruption of the inflammation–circadian axis may contribute to the rhythmic and metabolic alterations observed in the absence of functional GPNMB [46].

**FIGURE 7:**
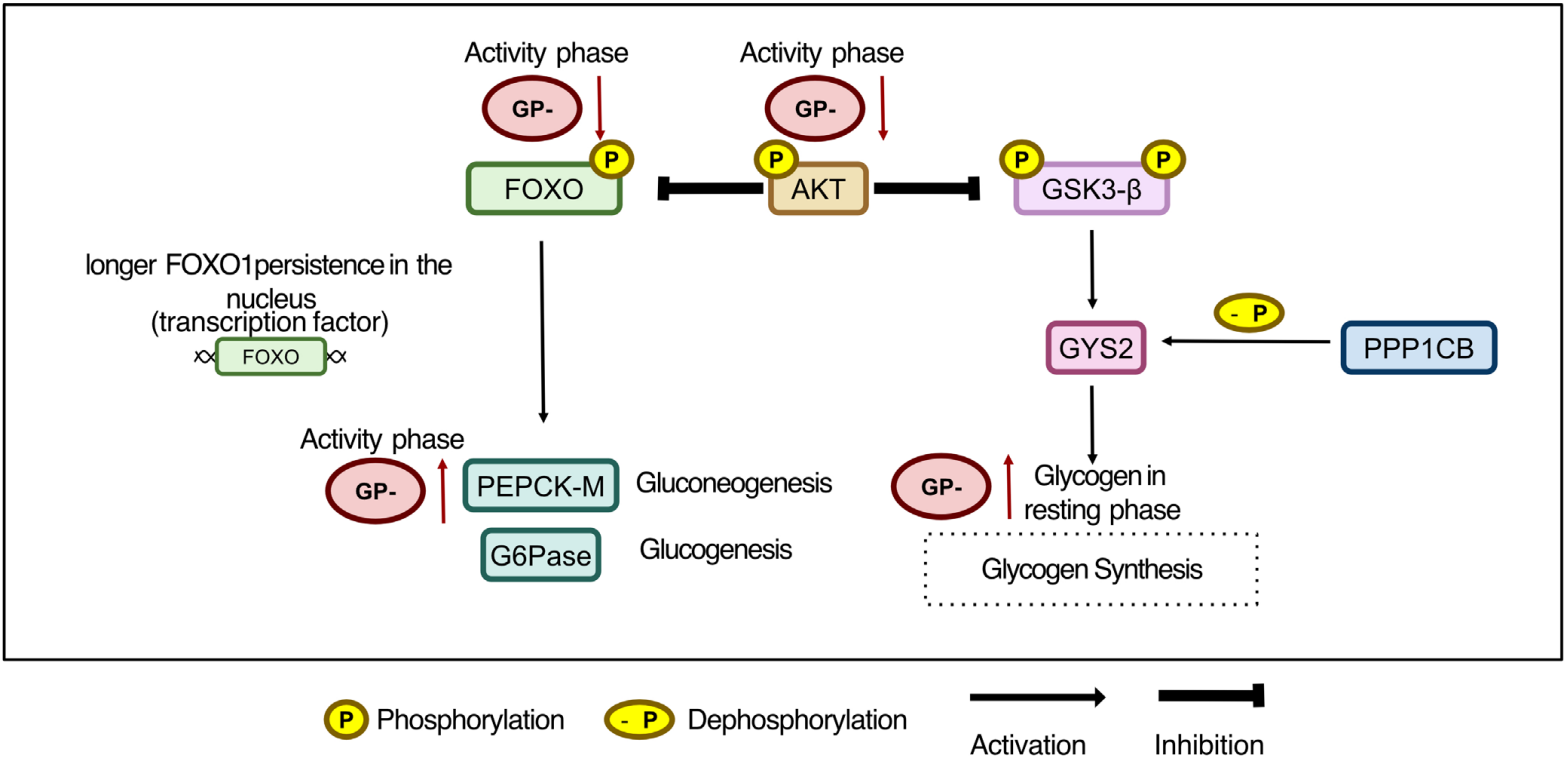
Schematic representation of hepatic insulin signaling in GPNMB‐deficient mice. Under physiological conditions, AKT phosphorylates and inhibits GSK3β, activating glycogen synthase (GYS2) and promoting hepatic glycogen accumulation. AKT also phosphorylates FOXO1, inducing its translocation from the nucleus to the cytoplasm and suppressing the transcription of gluconeogenic genes. In the absence of GPNMB (GP^−^), reduced AKT phosphorylation impairs insulin signaling, leading to increased transcriptional activity of FOXO1, upregulating PEPCK‐M in a time‐dependent manner. Despite this, hepatic glycogen content is elevated at the resting phase in GP− mice, suggesting defective glycogen mobilization. These findings indicate that GPNMB deficiency disrupts the balance between glycogen synthesis, utilization, and gluconeogenesis, thereby altering hepatic glucose homeostasis during the active phase.

Consistent with previous human data, our findings indicate that in human liver samples, increases in hepatic *GPNMB* expression correlate with the severity of MASLD. *GPNMB* expression was significantly higher in patients with MASH without DM2 compared to healthy obese individuals and MASL patients without DM2 (Figure 6). This pattern suggests that *GPNMB* is strongly associated with disease progression. In fact, we found a positive correlation between *GPNMB* and NAS score. This observation, together with previous literature [55], reinforces the notion that *GPNMB* expression is closely associated with hepatic inflammation during disease progression. Although previous studies have reported a positive correlation between plasma GPNMB and BMI [54], our data did not confirm this association (Table S3). This discrepancy may reflect differences in the tissue source of circulating GPNMB, as white adipose tissue represents a major source, particularly under metabolic stress and inflammatory conditions [30, 51]. Interestingly, patients with MASH and DM2 undergoing pharmacological treatment, predominantly with metformin, showed a significant reduction in *GPNMB* expression. These levels were similar to those observed in patients with MASL, with or without DM2, and in healthy obese individuals (Figure 6). This effect may be related to metformin's known hepatoprotective actions, which are mainly mediated by AMPK activation [72, 73]. This leads to reduced hepatic gluconeogenesis and the inhibition of lipogenesis via SREBP1 suppression. Metformin also stimulates fatty acid oxidation and modulates anti‐inflammatory and antifibrotic pathways [74, 75]. Thus, the reduction in *GPNMB* expression in these patients may result from metformin's ability to improve hepatic metabolic homeostasis and suppress pro‐inflammatory and lipotoxic pathways. Together, these findings reinforce the potential of *GPNMB* as a target protein for progression and therapeutic response in MASLD Figure 7.

## 5 Conclusion

Our findings indicate that GPNMB is associated with complex and tissue‐specific functions. In the context of diet‐induced metabolic stress, the absence of GPNMB is associated with a dissociation between glucose and lipid metabolism, characterized by increased body fat accumulation and lean mass loss (unaltered body weight), despite higher glycogen accumulation and glucose clearance. Such a complex scenario likely reflects interactions among multiple tissues, such as muscle and adipose tissue, which may affect the conclusions of our liver‐centric study. On the clinical side, our human data indicates that hepatic GPNMB expression is associated with metabolic regulation and is a sensitive target for diabetes treatment. The reduction in hepatic *GPNMB* observed in patients with MASH and TDM2 under anti‐diabetic therapy suggests that improved metabolic and inflammatory status downregulates its expression. This novel association is clinically relevant, as concomitant diabetes treatment in MASLD patients may improve liver health, thereby influencing *GPNMB* expression. Together, our results support the view that increased hepatic GPNMB is associated with obesity‐induced metabolic alterations and may reflect an adaptive response to metabolic stress.

## 6 Limitations

An important limitation of our study is the exclusive use of male mice. Previous findings showed that female GPNMB‐KO mice preserved insulin sensitivity and glucose tolerance comparable to WT controls even after HFD exposure [30]. Given that female mice are typically more resistant to diet‐induced obesity [30, 76], our results may not fully capture sex‐specific metabolic responses. Despite pronounced systemic alterations in glucose and lipid metabolism, the absence of a detailed hepatic phenotypic characterization limits the interpretation of a liver‐specific contribution. Although the use of coisogenic DBA/2 J‐Gpnmb model represents a valid form to account for putative genetic variation, the use of an isogenic line would be the most appropriate form of control to account for potential genetic variation. Although our data strongly support our claims associated with GPNMB protein, we cannot rule out slight effects associated with potential genetic variation between the mouse lines. Another limitation is that the use of a global GPNMB‐deficient model does not allow discrimination between direct hepatic effects and secondary systemic adaptations. Future studies using liver‐specific knockout models or rescue strategies would refine causal and organ‐specific mechanisms. Moreover, we cannot exclude adipose tissue or skeletal muscle involvement, as our analysis primarily focused on systemic metabolic parameters rather than tissue‐level mechanisms, potentially masking organ‐specific manifestations of GPNMB deficiency. Although clock gene expression was elevated in GP^−^ mice, we did not evaluate downstream transcriptional outputs of circadian regulation, preventing conclusions about whether the observed increase reflects enhanced clock strength. Also, glycogen and protein levels were assessed at only two time points; inclusion of a full diurnal profile would have provided a more comprehensive understanding of the temporal dynamics underlying GPNMB deficiency. Finally, although our experimental and clinical models differ substantially in nature, the former relying on GPNMB‐deficient mice under diet‐induced metabolic stress, and the latter reflecting chronic human MASLD progression, both provide complementary insights. Nonetheless, comparisons between mice and human data should be interpreted with caution, as the mouse model isolates the physiological consequences of global GPNMB loss under controlled conditions, whereas human liver samples capture adaptive or compensatory changes occurring during disease progression and pharmacological treatment.

## Author Contributions

de Assis LVM and Moraes MN: conceptualization. Furtado EMO: data curation, formal analysis. Caperuto L, Abreu MNS, Poletini MO, de Assis LVM, Moraes MN: furtado EM, formal analysis. Moraes MN; funding acquisition. Furtado EMO, Silva JJ, Tostes AF, Santos L, Barsanele PS, Santana‐Lima B, Jegodzinski L, Castven D, Schenk A: investigation. Furtado EMO, Silva JJ, Caperuto L, Soares DD: methodology. Moraes MN: project administration. de Assis LVM and Moraes MN: supervision. Furtado EMO; de Assis LVM and Moraes MN: visualization. Furtado EMO; Caperuto L; Poletini MO; de Assis LVM; Moraes MN: writing – original draft. All authors; writing – review and editing. All authors have approved the final version of the manuscript and have agreed to take full responsibility for all aspects of the study, ensuring that any issues relating to the accuracy or integrity of any part of the work are properly investigated and resolved. All individuals listed as authors meet the criteria for authorship, and there are no exclusions of people who also meet these criteria.

## Funding

This work was partially supported by the São Paulo Research Foundation (FAPESP grants 2017/26651–9 and 2022/15729–5 Moraes MN), and the National Council of Technological and Scientific Development (CNPq) grant 406 445/2023–0 (Poletini MO, Soares DD, de Assis LVM, Moraes MN). Furtado EMO (2023/08461–9) and Barsanele PS (2022/07969–6) were fellows of FAPESP; Silva JJ (88887.663829/2022–00), Santos L (88887.919365/2023–00), and Santana‐Lima B (88887.837807/2023–00) are fellows of Capes, and Tostes AF (161 437/2022–2) is a fellow of CNPq. de Assis LVM is supported by the Knut and Alice Wallenberg Foundation as a Wallenberg Molecular Medicine Fellow and by the German Research Foundation (Deutsche Forschungsgemeinschaft) grant 541 063 275—TRR 418 B02 (Marquardt) and B04 (de Assis).

## Conflicts of Interest

The authors declare no conflicts of interest.

## Supporting information


**Data S1:** fsb271616‐sup‐0001‐Supinfo.pdf.

## Data Availability

Data are available upon request to the corresponding author.
