# Supplementary material for: Lack of GPNMB Is Associated With Altered Lipid and Glucose Metabolism and Disrupted Diurnal Hepatic Glycogen Regulation
Source: FASEB J. 2026 Mar 9;40(6):e71616. doi: 10.1096/fj.202504363R (PMC12969540; doi:10.1096/fj.202504363R)
Supplement: Supplementary file 1 — Data S1: fsb271616‐sup‐0001‐Supinfo.pdf. [file FSB2-40-e71616-s001.pdf]

SUPPLEMENTAL DATA

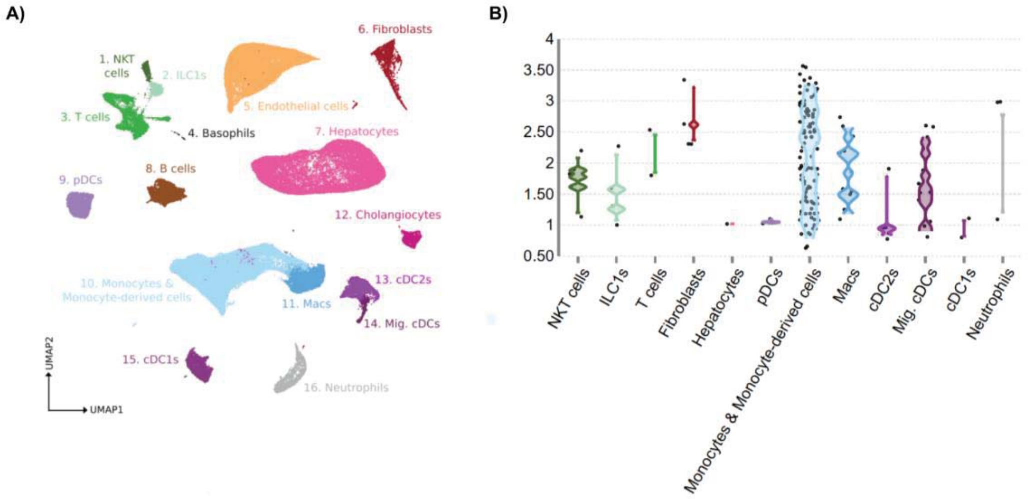

Supplementary Figure 1. Mapping of *Gpnmb* expression in the cellular heterogeneity of liver tissue in mice with MASLD (Metabolic dysfunction-Associated Steatotic Liver Disease). (A) UMAP (Uniform Manifold Approximation and Projection) analysis of the distribution of different hepatic cell populations, based on the gene expression of individual cells. (B) Graphs in violin plot format illustrate the *Gpnmb* gene expression distribution in different hepatic cell types. Each color represents a distinct cell type. The data were extracted from the Liver Cell Atlas database (https://www.livercellatlas.org/umap-NAFLDmouseAll.php).

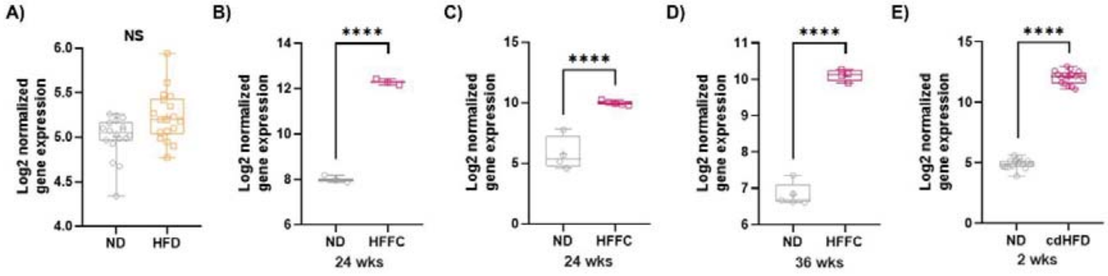

Supplementary Figure 2. Expression of *Gpnmb* is not increased in mice fed with HFD but increases in mice fed with MASH-inducing diets. RNAseq data were obtained from three independent studies through the GEO database and processed by the DESeq2 tool for differential expression analysis. Student's t-test analyzed microarray data. In both cases, the p-value was corrected by the false discovery rate method (FDR < 0.01). Differential expression of *Gpnmb* in the liver of animals subjected to HFD from databases deposited by (a) Eckel-Mahan et al., 2013; (b) Furuta et al., 2021; (c) Lee et al., 2021; (d) Ægidius et al., 2021; (e) de Assis et al., 2023. Genes were considered differentially expressed when the p-value was <0.01 and FC±1 (log2 values).

Supplementary Table 1. Rhythmic parameters of RER and EE of GP+ and GP- mice in a thermoneutral zone (30ºC) after 8 and 12 weeks of HFD+FRUT

	Paramiter	Presence of rhythmicity (p-value)		Amplitude			Mesor			Acrophase (ZT - hours)		
		GP+	GP-	GP+	GP-	p-value	GP+	GP-	p-value	GP+	GP-	p-value
8 weeks	O2	**0.0109**	**3.01E-05**	6.1370	5.6918	0.8677	38.0342	28.5485	**1.51E-06**	18.82	16.96	0.2831
	CO2	**0.0084**	**9.06E-05**	6.5504	4.7756	0.5077	35.6169	25.4403	**3.03E-07**	19.12	17.10	0.2828
	RER	**0.0232**	**0.0170**	0.0202	0.0133	0.5071	0.9231	0.8924	**4.18E-05**	19.98	4.17	**0.0013**
	EE	**0.0103**	**3.76E-05**	0.1313	0.1156	0.7802	0.7912	0.5872	**9.84E-07**	18.90	16.99	0.2759
12 weeks	O2	**0.0201**	0.1270	5.9385	3.7713	0.5358	41.3020	30.1097	**1.25E-05**	17.76	**NR**	-
	CO2	**0.0151**	0.1316	5.8830	3.3608	0.4360	37.6391	26.7666	**4.77E-06**	17.74	**NR**	-
	RER	**0.0007**	0.0696	0.0276	0.0123	0.1379	0.9064	0.8854	**0.0043**	17.38	**NR**	-
	EE	**0.0181**	0.1283	0.1260	0.0774	0.5032	0.8544	0.6189	**9.34E-06**	17.75	**NR**	-

Supplementary Table 2. Rhythmic parameters of gene expression in the liver of GP+ and GP- mice subjected to 12 weeks of HFD+FRUT.

Gene Expression	Presence of rhythmicity (p-value)		Amplitude			Mesor			Acrophase (ZT - hours)		
	GP+	GP-	GP+	GP-	p-value	GP+	GP-	p-value	GP+	GP-	p-value
Bmal1	**8.09E-09**	**4.34E-10**	5.35116	6.17192	0.38481	6.19725	5.99429	0.74098	22.28	21.25	0.06100
Per1	**3.03E-07**	**3.35E-06**	5.74940	9.31089	0.06001	6.88734	10.03753	**0.01427**	11.79	10.84	0.32593
Per2	**1.60E-07**	**0.00035**	2.46156	1.86095	0.30614	3.49470	4.66015	**0.01016**	15.86	17.39	0.20726
Nr1d1	**0.00022**	**0.00002**	27.01496	52.45811	**0.04487**	19.80005	36.34463	**0.05682**	7.41	6.54	0.51862
Slc2a2	0.87931	**0.03165**	0.02183	0.27229	0.18572	1.20156	1.15420	0.72121	**NR**	15.17	-
Gys2	**0.03227**	**0.00302**	0.15995	0.26185	0.34998	1.21953	1.24697	0.73917	15.77	14.37	0.57553
Ppar-α	0.09991	**0.00034**	0.33339	0.85108	0.07698	1.14766	1.40186	0.17995	**NR**	9.81	-
Ppar-γ	0.42014	0.11134	0.19022	0.30067	0.71049	1.58182	1.14444	**0.03596**	**NR**	**NR**	-
Hsl	**0.03868**	**0.00025**	0.61004	1.14248	0.18045	1.76458	2.11740	0.18612	11.35	11.29	0.97264
Scap	0.70886	0.66037	0.07865	0.09376	0.95985	1.14855	1.42664	0.21114	**NR**	**NR**	-
Tnf-α	0.11201	0.63344	0.33165	0.13587	0.57912	1.31244	1.56657	0.27287	**NR**	**NR**	-
Il6r-α	**0.00145**	**0.00009**	0.74780	1.03843	0.35335	1.72628	2.25675	**0.02248**	14.08	12.80	0.39662

Supplementary Table 3. Bivariate analysis evaluating the correlation between the numerical explanatory variables and the *GPNMB* expression

	Correlation	p-value
Age	0.278*	0.1
BMI	0.100*	0.57
Albumin	-0.27	0.111
Uric acid	0.092	0.593
TSH	-0.119	0.488
Cholesterol	-0.008*	0.962
LDL	0.022	0.904
SREBP1 mRNA	-0.199	0.243
FOXO1 mRNA	**0.348****	**0.041**
FBP1 mRNA	0.141	0.411
TGO-AST	0.156	0.364
TGP-ALT	0.089	0.604
GammaGT	0.139	0.419
Fib4	0.153	0.372
Creatinine	0.258	0.129
Glycated hemoglobin	0.309	0.067
HDL	0.022	0.899
Pathology NAS-Score	**0.500****	**0.002**

*Pearson correlation coefficient
**Spearman's correlation coefficient

Supplementary Table 4. Bivariate analysis evaluating the initial diagnosis, the presence of diabetes, and the medications used with the *GPNMB* expression

	Mean	Standard deviation	p-value
Diagnosis			
HO	1.56	1.91	
MASL	3.63	1.01	**0.001***
MASH	3.7	1.16	
Diabetes			
No	2.62	1.55	**0.043****
Yes	3.8	1.63	
ACEAT2RHemmer			
No	2.7	1.63	0.179**
Yes	3.46	1.63	
Beta blocker			
No	2.85	1.68	0.382**
Yes	3.38	1.62	
CA_Antagonists			
No	2.95	1.82	0.604**
Yes	3.21	1.05	
Statins			
No	2.92	1.62	0.631**
Yes	3.21	1.79	
ASS			
No	3	1.7	0.520**
Yes	3.19	0	
Allopurinol			
No	2.99	1.69	0.767**
Yes	3.36	1.34	
LThyroxin			
No	2.97	1.66	0.694**
Yes	3.29	1.77	
Insulin			
No	3.06	1.69	0.636**
Yes	2.57	1.38	
Metformin			
No	2.76	1.44	0.143**
Yes	3.67	2.07	
Ozempic			
No	3.04	1.74	0.845**
Yes	2.89	1.25	

***ANOVA **Student's t-test**
